# Direct removal of RNA polymerase barriers to replication by accessory replicative helicases

**DOI:** 10.1093/nar/gkz170

**Published:** 2019-03-14

**Authors:** Michelle Hawkins, Juachi U Dimude, Jamieson A L Howard, Abigail J Smith, Mark S Dillingham, Nigel J Savery, Christian J Rudolph, Peter McGlynn

**Affiliations:** 1 Department of Biology, University of York, Wentworth Way, York YO10 5DD, UK; 2 Division of Biosciences, College of Health and Life Sciences, Brunel University London, Uxbridge UB8 3PH, UK; 3 DNA-Protein Interactions Unit, School of Biochemistry, University of Bristol, Bristol BS8 1TD, UK

## Abstract

Bacterial genome duplication and transcription require simultaneous access to the same DNA template. Conflicts between the replisome and transcription machinery can lead to interruption of DNA replication and loss of genome stability. Pausing, stalling and backtracking of transcribing RNA polymerases add to this problem and present barriers to replisomes. Accessory helicases promote fork movement through nucleoprotein barriers and exist in viruses, bacteria and eukaryotes. Here, we show that stalled *Escherichia coli* transcription elongation complexes block reconstituted replisomes. This physiologically relevant block can be alleviated by the accessory helicase Rep or UvrD, resulting in the formation of full-length replication products. Accessory helicase action during replication-transcription collisions therefore promotes continued replication without leaving gaps in the DNA. In contrast, DinG does not promote replisome movement through stalled transcription complexes *in vitro*. However, our data demonstrate that DinG operates indirectly *in vivo* to reduce conflicts between replication and transcription. These results suggest that Rep and UvrD helicases operate on DNA at the replication fork whereas DinG helicase acts via a different mechanism.

## INTRODUCTION

Genome duplication and transcription require access to the same template DNA. These access requirements create a potential conflict between two large multi-subunit enzyme complexes that could lead to interruption of DNA replication and consequently a possible loss of genome stability (1–4). Pausing, stalling and backtracking of transcribing RNA polymerases, either on undamaged or damaged template DNA, compound this problem (5,6). Such immobile RNA polymerases present static and long-lived barriers to replisomes.

Transcription elongation and termination factors can reduce the number of immobile RNA polymerases on DNA. For example, the double-stranded DNA translocase activity of *Escherichia coli* Mfd targets paused and stalled RNA polymerases resulting in either resumption of transcription or displacement of the stalled RNA polymerase (7,8). The formation of RNA-DNA hybrids between the transcript and the template DNA strand (R-loops), may also present problems for replisomes when collisions are head-on (9–11). Whether R-loops present problems directly for replisome progression or merely stabilize the transcription complex is unknown. Regardless of the exact nature of the barrier caused by R-loops, ribonucleases specific for RNA-DNA hybrids that can degrade R-loops are ubiquitous (4) which indicates that they are a significant source of problems. Replication and transcription conflicts are also important in eukaryotes and R-loops can cause problems for the eukaryotic replisome. While eukaryotic replication and transcription are largely separated via the cell cycle, some genes are still transcribed during S phase. In humans the Pif1 helicase family or SETX helicases limit R-loop accumulation (12). Defects in these mechanisms have been associated with breast cancer and neurological pathologies respectively (12).

In spite of all these conflict reduction mechanisms, replisomes do still encounter transcriptional barriers in head-on and co-directional orientations. Early *in vitro* evidence demonstrated that head-on transcription led to severe inhibition of replication fork progression while co-directional transcription did not appear to have an effect (13). Given that essential and highly transcribed genes are often encoded on the leading strand to ensure co-directional transcription and replication, head-on collisions are thought to be more deleterious. However, co-directional collisions of the replisome and transcription machinery also impact replication fork progression *in vivo* (14). The replisome itself can disrupt transcription complexes. *E. coli* replisomes halt at a stalled RNA polymerase *in vitro* and can continue replication through stalled transcription complexes in both co-directional and head-on orientations, although the replisome pauses for far longer in the head-on as compared with the co-directional orientation (15,16). Furthermore, after displacement of the RNA polymerase in co-directional, but not head-on, collisions the *E. coli* replisome can use the RNA transcript to re-prime leading strand synthesis (15). However, the efficiency of this disruption must be insufficient to allow rapid genome duplication and the maintenance of genetic stability *in vivo* since specific mechanisms are needed to aid replisome movement during such collisions (4,17).

Accessory replicative helicases promote fork movement through nucleoprotein barriers and have been identified in both prokaryotes and eukaryotes (18–22). These accessory helicases reduce replisome pausing at many different types of nucleoprotein complex *in vivo*, including transcription complexes (18,20,21). The *E. coli* accessory helicase Rep can promote movement of the *E. coli* replisome through a model nucleoprotein barrier *in vitro*, namely a mutant restriction enzyme that can bind to but not cleave its cognate site (19). Protein displacement from DNA ahead of the replication fork by Rep likely involves translocation 3′-5′ along the leading strand DNA template at the fork, opposite that of the primary replicative helicase DnaB 5′-3′ along the lagging strand template (23).

Two other *E. coli* helicases, UvrD and DinG, also act *in vivo* to reduce conflicts between replication and transcription (19,20). UvrD, a homologue of Rep that also translocates 3′-5′ along ssDNA, (24,25) can compensate partially for the absence of Rep *in vivo* (20,26–28). UvrD can also promote replisome movement through model nucleoprotein barriers *in vitro* (19). However, minimisation of replication fork pausing at nucleoprotein complexes in wild type cells requires Rep, not UvrD (29). The ability of UvrD to compensate partially for the absence of Rep has been attributed to the high degree of homology between these two helicases and the abundance of UvrD inside cells (19). UvrD also interacts directly with RNA polymerase (30–32), an interaction that has been suggested to promote backtracking of stalled RNA polymerase as a first step in transcription-coupled repair (31). However, other studies argue against UvrD playing any role in coupling nucleotide excision repair to stalled transcription complexes (33–35). The function of this UvrD-RNA polymerase interaction remains unclear. In *Bacillus subtillus* the essential UvrD/Rep homologue PcrA also interacts with RNA polymerase (30) and has been shown to promote replication through transcribed genes (36).

DinG helicase interacts functionally with Rep and UvrD *in vivo*, indicating that DinG might also promote duplication of transcribed DNA (20). Genetic evidence also suggests that DinG might inhibit the formation and/or promote the removal of R-loops (20). However, DinG is a 5′-3′ helicase unrelated to Rep and UvrD (37,38) and this 5′-3′ polarity makes it unlikely that promotion of fork movement by DinG occurs via the same mechanism as either Rep or UvrD (23). Furthermore, unlike Rep and UvrD, there are no data indicating that DinG promotes replication fork movement through model nucleoprotein barriers *in vitro*.

Here, we establish that *E. coli* transcription elongation complexes stalled by nucleotide deprivation can block reconstituted replisomes. Blockage can be alleviated by either Rep or UvrD, promoting the formation of full-length replication products. Thus accessory helicase action during replication-transcription collisions promotes continued replication without leaving gaps in the DNA. This contrasts with the re-priming needed when a replisome itself overcomes a stalled transcription complex in the co-directional orientation (15). Unlike Rep and UvrD, DinG cannot promote replisome movement through stalled transcription complexes *in vitro*. These data demonstrate that Rep and UvrD can promote replisome movement through transcription complexes directly, in agreement with evidence that Rep is the accessory replicative helicase in wild type cells and that UvrD can compensate for the absence of Rep. Furthermore, the ability of Rep and UvrD to promote replication fork movement through two very different types of nucleoprotein complex, namely transcription complexes as shown here and catalytically-inactivated EcoRI endonuclease (19), implies that they may assist with bypass of many different types of nucleoprotein complexes *in vivo*. In contrast, our data indicate that any promotion of replication of transcribed DNA by DinG occurs via a mechanism that does not involve displacement of RNA polymerases ahead of an advancing replication fork.

## MATERIALS AND METHODS

### Plasmids, proteins and strains

pPM854 was formed by ligating the 4.4 kb EcoRI-EagI fragment from pBROTB535-I (39) with the 0.9 kb EcoRI–EagI fragment from pBR322. To form pPM872 and pPM875, two plasmid constructs were synthesized by Eurofins MWG Operon. pEX-A-UV5 plus *bla* had P_lacUV5 52C_ fused to the 5′ end of the *bla* gene whilst *pEX-A-UV5* plus *tet* had P_lacUV5 52C_ fused to the 5′ end of *tet* (see Figure 2B). P_lacUV5 52C_ was derived from pSRc33 (40). Using site-directed mutagenesis all the C residues between +1 and +52 on the non-transcribed strand were removed. This enables transcription of P_lacUV5 52C_ to be stalled by the omission of CTP. The EcoRI-ScaI fragment from *pEX-A-UV5* plus *bla* was then cloned into EcoRI-ScaI cut pPM854 to generate pPM870. A filler fragment was then added to pPM870 by taking the 3′ end of *rep* as an EcoRV fragment from pCC141, a pBluescript II SK(-) plasmid containing *rep*, and cloning into the EcoRV site of pPM870 to form pPM872. To form pPM875, the EcoRI-EcoRV fragment from pEX-A-UV5 plus *tet* was cloned into EcoRI-EcoRV cut pPM854 to generate pPM868. The EcoRV fragment from pCC141 was then cloned into the ScaI site of pPM868. Partial sequence from the kanamycin^R^ cassette was used to generate modified versions of pPM872 with additional promotor-free DNA inserted either side of *oriC*. MscI sites were added to 0.5 kb of internal kanamycin^R^ cassette sequence (41) using oMH046/70 and cloned into the MscI site of pPM872 to construct pMH033. PCR with oMH046/49 was used to add *terB* to internal kanamycin^R^ cassette sequence and this fragment was cloned into the NdeI site of pUC19. The 0.6 kb *terB*-kan NdeI fragment was then cloned into the NdeI site of pPM872 to construct pMH049.

Purification of replication assay proteins was performed as described in (29). Rep and UvrD were purified as described in (42). Mfd was purified as described in (43). DinG was a kind gift from Daniel Camerini-Otero (37). Protein concentration was determined by Bradford assay according to manufacturer instructions (Sigma). None of the proteins used in this study display detectable nuclease activity on our substrates over the timescale of these experiments.

Please see Supplementary Table S2 for strains and construction details.

### Replication assay

**Figure 1.**
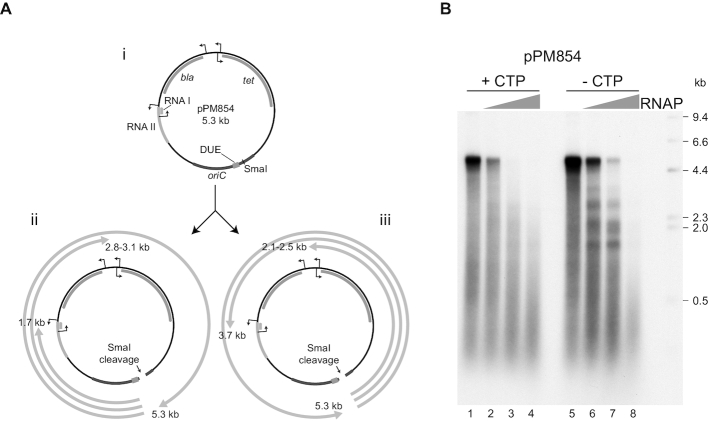
Inhibition of DNA replication by RNA polymerase. (**A**) Diagram of pPM854 (i) indicating known promoters and their associated open reading frames. RNAI and RNAII are the two open reading frames that form the *ColE1* plasmid origin of replication. The cloned chromosomal origin of replication *oriC*, the duplex unwinding element (DUE) where strand opening initiates within *oriC* and the *Sma*I restriction site used to allow replication elongation to proceed in the absence of a topoisomerase are also indicated. Distances from the site of replication initiation within *oriC* to the transcription promoters are indicated for the clockwise (ii) and counter-clockwise (iii) moving forks. (**B**) Denaturing agarose gel of replication products formed with pPM854 in the absence or the presence of increasing concentrations of RNA polymerase with and without CTP being present. Marker sizes in kilobases are indicated. Lagging strand replication products migrate near the 0.5 kb marker whilst the majority of leading strand products are close to the full-length of 5.3 kb in the absence of RNA polymerase (lanes 1 and 5).

Assays were performed in 40 mM HEPES (pH 8); 10 mM DTT; 10 mM magnesium acetate; 2 mM ATP; 0.2 mM GTP, CTP and UTP in standard reactions or with omission of CTP in reactions where RNA polymerase was stalled; 0.04 mM dNTPs; and 0.1 mg/ml BSA. Reactions (15 μl) contained 2 nM plasmid template, 50 nM DNA polymerase III αϵθ complex, 25 nM τ clamp loader complex, 160 nM DnaB and DnaC monomers, 1 μM SSB, 80 nM beta clamp, 30 nM HU, 200 nM DnaG, 300 nM DnaA. Helicases were added as indicated; Rep and UvrD at 200 nM final concentration, Mfd and DinG at 100 nM final concentration. *E. coli* RNA polymerase holoenzyme (1 U/μl) was from Epicentre (Figures 1 and 3) or Affymetrix (Figures 4, 5 and Supplemental Figure S1) and used at ¼ dilution (1 μl/reaction, 20 nM final concentration). Equivalent RNA polymerase replication inhibition was determined empirically as suppliers were switched. Reactions were assembled on ice and initiated by addition of DnaA and incubation for 4 minutes at 37°C, followed by addition of 60 units SmaI (Promega, high concentration) plus 0.4 MBq [α**^32^**P] dCTP (222 TBq/mmol). Reactions were carried out at 37°C for 1 min and then terminated by addition of 1 μl of 0.5 M EDTA. Replication products were analysed by denaturing agarose gel electrophoresis (0.7% agarose in 2 mM EDTA 30 mM NaOH for 400 Vh, standard run was 16 h at 25 V), phosphorimaging and autoradiography. 5′-Labelled HindIII-digested λ DNA was used as a marker.

### Transcription assay

**Figure 2.**
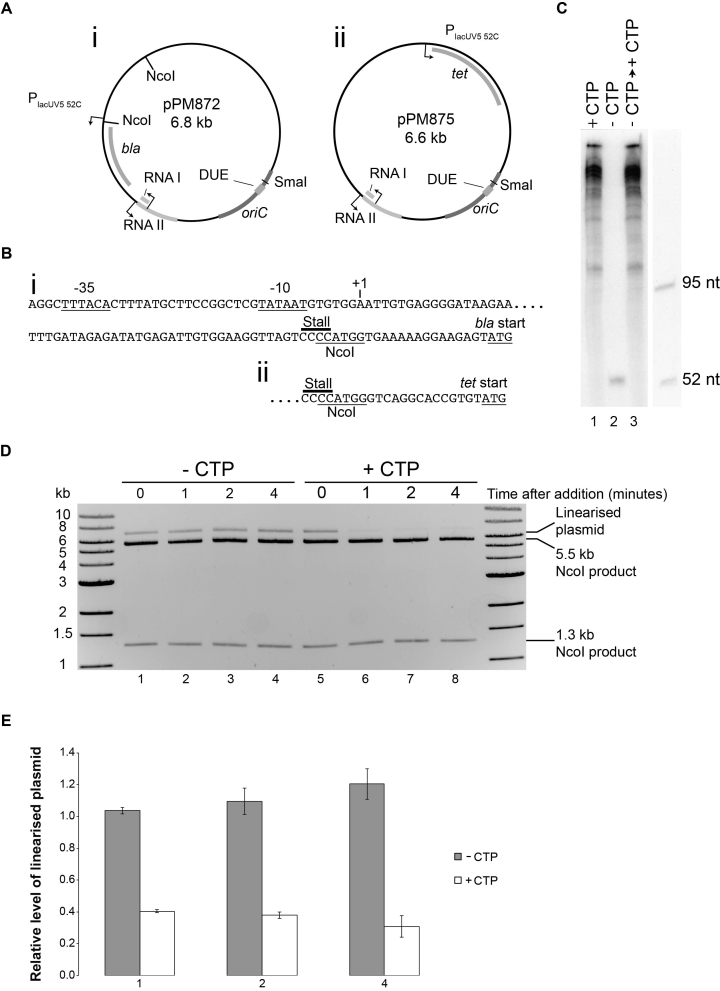
Engineering a stalled transcription elongation complex. (**A**) Maps of pPM872 and pPM875. (**B**) Sequences of P_lacUV5 52C_ upstream of (i) *bla* and (ii) *tet* in pPM872 and pPM875, respectively. The –35 and –10 sequences of the promoter, the transcription start site, the stall site and the NcoI restriction site are all shown together with the start codon of each gene. Note that the native sequences upstream of each gene were used in between the NcoI site and the start codon. (**C**) Denaturing gel of RNA products formed by RNA polymerase on pPM872 in the presence of all four ribonucleotides (lane 1) and upon omission of CTP (lane 2). Transcripts were also analysed after incubation in the absence of CTP followed by subsequent addition of CTP (lane 3). (**D**) The accessibility of the NcoI restriction site within P_lacUV5 52C_ in pPM872 was monitored using native agarose gel electrophoresis. RNA polymerase was added to pPM872 in the absence of CTP in two parallel reactions and incubation continued for four minutes. A sample was removed at time zero (lanes 1/5) and cleaved with NcoI. Immediately after the sample was removed, water or CTP was added and then cleavage by NcoI analysed 1, 2 and 4 min after addition of water (lanes 2–4) or CTP (lanes 6–8). Cleavage at both NcoI sites generated 1.3 kb and 5.5 kb fragments whereas cleavage at just one of the NcoI sites in pPM872 generated linearized plasmid of 6.8 kb. (**E**) Relative levels of linearized plasmid compared to completely cut plasmid with respect to the zero time point. Error bars represent ± one standard error of three experiments.

For plasmid templates 177 ng of SmaI linearized pPM872 was used per reaction. RNA polymerase (Affymetrix in Figure 2, NEB in Supplemental Figure S2) was added to a final concentration of 20 nM and incubated for 15 min at 37°C in 40 mM HEPES (pH 8); 10 mM DTT; 10 mM magnesium acetate, 0.1 mg/ml BSA. Transcription assays were carried out in 40 mM HEPES (pH 8); 10 mM DTT; 10 mM magnesium acetate, 0.1 mg/ml BSA to match replication assay conditions with 2 mM ATP, 10 μM GTP, 200 μM UTP, 40 μM dNTPs, 0.4 MBq [α**^32^**P] GTP (222 TBq/mmol) and 200 μM CTP added simultaneously if indicated. Reactions were assembled on ice and carried out at 37°C for 5 minutes before termination with an equal volume of stop mix (7 M urea, 10 mM EDTA, 1% SDS, 2× TBE, 0.05% bromophenol blue, 0.05% xylene cyanol). To test for post-stall transcription, reactions lacking CTP were incubated for 5 minutes at 37°C before CTP addition to a final concentration of 200 μM. The reaction was then allowed to proceed for a further 5 minutes at 37°C before termination. Transcription products were purified on P-6 Microbiospin columns and run on a 15% acrylamide 7 M urea gel before phosphorimaging and autoradiography analysis. T4 polynucleotide kinase labeled oligonucleotides oMH099 (95 nt), oMH111 (52 nt) were used as markers.

### RNA polymerase occupancy assay

20 nM RNA polymerase was added to pPM872 in the presence of 200 nM Rep, UvrD, Mfd or with no helicase. Reactions were performed in replication assay conditions (40 mM HEPES pH 8; 10 mM DTT; 10 mM magnesium acetate; 2 mM ATP; 0.2 mM GTP, and UTP, 0.04 mM dNTPs; and 0.1 mg/ml BSA but without replication proteins or CTP) and incubated at 37°C for 4 minutes. A 15 μl sample was removed and cleaved with 20 units of pre-warmed NcoI-HF (NEB) at 37°C for 90 s. Immediately after the sample was removed, CTP or dH_2_O was added to this reaction (200 μM final concentration CTP) and cleavage by NcoI was analysed 1, 2 and 4 minutes after addition of CTP as above. Cleavage was stopped by addition of 1 μl 0.5 M EDTA and heat inactivation (80°C for 10 minutes). Products were analysed by native agarose gel electrophoresis.

### Helicase assay

Helicase assays were performed as previously described (44). Unwinding of forked substrates ± streptavidin was carried out in 10 μl volumes of 40 mM HEPES (pH 8); 10 mM DTT; 10 mM magnesium acetate; 2 mM ATP; 0.1 mg/ml BSA and 1 nM forked DNA substrate. Reactions were incubated for 5 minutes at 37°C ± streptavidin. DinG and biotin were added and reactions allowed to continue for a further 10 minutes at 37°C. Reactions were stopped with the addition of 2.5 μl of 2.5% SDS, 200 mM EDTA and 10 mg/ml of proteinase K and analysed by non-denaturing gel electrophoresis on 10% polyacrylamide gels. See the Supplementary Methods section for a more detailed description.

### Marker frequency analysis by deep sequencing

Marker frequency analysis by deep sequencing was performed as described previously (45). Fresh overnight cultures were diluted 100-fold in LB broth and incubated with vigorous aeration until *A*_600_ reached 0.48 at 37°C. Cultures were diluted again 100-fold in pre-warmed fresh broth and grown until *A*_600_ = 0.48. Samples were flash-frozen in liquid nitrogen for subsequent DNA extraction. For a wild type stationary phase sample, incubation of the remaining culture was continued for several hours post-saturation and a further sample was then frozen. DNA was extracted using the GenElute Bacterial Genomic DNA Kit (Sigma-Aldrich). Marker frequency analysis was performed using Illumina HiSeq 2500 sequencing (fast run) to measure sequence copy number. The enrichment of uniquely mapped sequence tags in 1 kb windows was calculated. Replication profiles of all key constructs were confirmed by two independent experiments. See the Supplementary Methods section for a more detailed description.

### Synthetic lethality assay

The synthetic lethality assay was performed as previously described (46,47). Strains carrying *dinG*^+^ derivatives of pRC7 (a *lac+* mini-F plasmid that is rapidly lost) were grown in LB broth with 100 μg/ml of ampicillin overnight, diluted 100-fold into LB broth without ampicillin and grown to *A*_600_ = 0.4. Dilutions were plated onto LB or M9 glucose minimal salts agar plates containing 120 μg/ml X-gal and 1 mM IPTG. Plates were photographed and scored after 48 h (LB agar) or 72 h (M9 agar) at 37°C. See the Supplementary Methods section for a more detailed description.

## RESULTS

### *In vitro* replication is inhibited by collisions with transcription elongation complexes

*E. coli* replisomes can be reconstituted on plasmid DNA templates containing *oriC*. In the absence of a topoisomerase only one of the two replication forks originating from *oriC* progresses to any significant extent and even this fork can proceed only ∼1 kb due to the accumulation of positive supercoiling (48). Subsequent cleavage of the template DNA with a restriction enzyme relieves this positive torsional strain and allows this fork to proceed around the template (49). Using an *oriC*-containing pBR322 plasmid, pPM854, as a replication template (Figure 1Ai) we used SmaI to relieve positive torsional strain and allow replication fork progression. Fork progression generated leading strand products of approximately 5 kb plus a smear of lagging strand products centred around 0.5 kb (Figure 1Aii and iii; Figure 1B, lane 1). Addition of RNA polymerase in the presence of all four ribonucleotides resulted in inhibition of full-length leading strand products and a concomitant decrease in levels of incorporation (Figure 1B, lanes 1–4). This inhibition did not result in distinct truncated leading strands indicating that inhibition of replication fork movement occurred in many different locations around the template. There are several well-characterized promoters within the pBR322 backbone (Figure 1A) in addition to any spurious transcription initiation that might occur elsewhere. It is also possible that any transcription across the *oriC* fragment could have prevented replication initiation via disruption of DnaA binding.

To prevent RNA polymerases from entering the elongation phase of transcription we omitted CTP from the reaction to stall RNA polymerases at the first guanine residue encountered within the transcription template strand. Note that DnaG primase can still synthesize ribonucleotide primers for DNA replication in the absence of CTP (50). Omission of CTP again resulted in inhibition of full-length leading strand products upon addition of RNA polymerase (Figure 1B, lanes 5–8). Since omission of CTP was predicted to stall RNA polymerases within at most a few nucleotides of transcription prior to formation of a stable elongation complex (51), these data indicate that promoter-proximal complexes present challenges to replisome movement. It is also possible that promoter-bound RNA polymerase acts as a replisome barrier. At lower concentrations of RNA polymerase more distinct truncated leading strands were produced as compared with reactions in which all four ribonucleotides were present (Figure 1B, compare lanes 3 and 7). However, it was still difficult to ascribe specific truncated leading strand products to individual transcription units. Furthermore, any misincorporation or contamination of ATP, GTP and UTP preparations with CTP would allow transcription to proceed beyond the first guanine residue encountered on the template strand.

### Co-directional and head-on collisions of replisomes and stalled transcription elongation complexes lead to halting of replication forks

We redesigned the replication template to reduce the number of native promoters and introduced a well-characterized strong promoter with a specific RNA polymerase stall site to facilitate formation of a stalled transcription elongation complex. A *lacUV5* promoter was used in which the first 52 nucleotides of the transcript lacked cytosine residues but were then followed by four consecutive cytosines, providing a very efficient transcription stall site in the absence of CTP (Figure 2B). The original promoters for the *bla* and *tet* genes within pPM854 were deleted and P_lacUV5 52C_ placed either upstream of the *bla* gene (pPM872) or upstream of the *tet* gene (pPM875) (Figure 2A). Transcription of pPM872 under replication assay conditions generated multiple long transcripts in the presence of CTP but only a single transcript of approximately 52 nucleotides in the absence of CTP, as expected from the P_lacUV5 52C_ promoter (Figure 2C, compare lanes 1 and 2). We also assessed the degree of occupancy of this stall site at concentrations of RNA polymerase that gave partial inhibition of replication (Figure 1B, lanes 2 and 6). We exploited an NcoI restriction site engineered to overlap with the stall site (Figure 2B). RNA polymerase stalling at this position should prevent cleavage of this restriction site by NcoI, resulting in linearization of the plasmid at the second NcoI site within the plasmid as opposed to cleavage into two DNA fragments (Figure 2D and 2Ai). In the absence of CTP ∼34% of the plasmid template was linearized upon addition of NcoI, indicating that a third of the template DNA contained RNA polymerase stalled at P_lacUV5 52C_ (Figure 2D, lanes 1–4). This RNA polymerase stall remained stable over the 4 minute duration of the pre-incubation step in the replication assay (Figure 2D and E). RNA polymerase occupancy was also unchanged after 60 minutes of stall conditions (Supplementary Figure S1B and 1C) so this block can be considered chronic. We also probed whether RNA polymerase stalled at this site was in a stable backtracked state by exploiting the inability of such backtracked complexes to resume transcription upon addition of the missing nucleotide (52). The transcript generated in the absence of CTP disappeared upon subsequent addition of CTP and longer length transcripts appeared (Figure 2C, lane 3). Thus the stalled RNA polymerase was not in a stable backtracked state. Furthermore, the linearized plasmid generated by cleavage of only a single NcoI site in pPM872 in the absence of CTP disappeared upon subsequent addition of CTP, indicating that stalled RNA polymerase was no longer occluding the NcoI site downstream of P_lacUV5 52C_ (Figure 2D, lanes 5–8). This stall release occurred within the first minute after addition of CTP and no further large reductions of linearized plasmid were observed within four minutes (Figure 2E). These data support the conclusion that the stalled RNA polymerase was not in a stable backtracked state.

**Figure 3.**
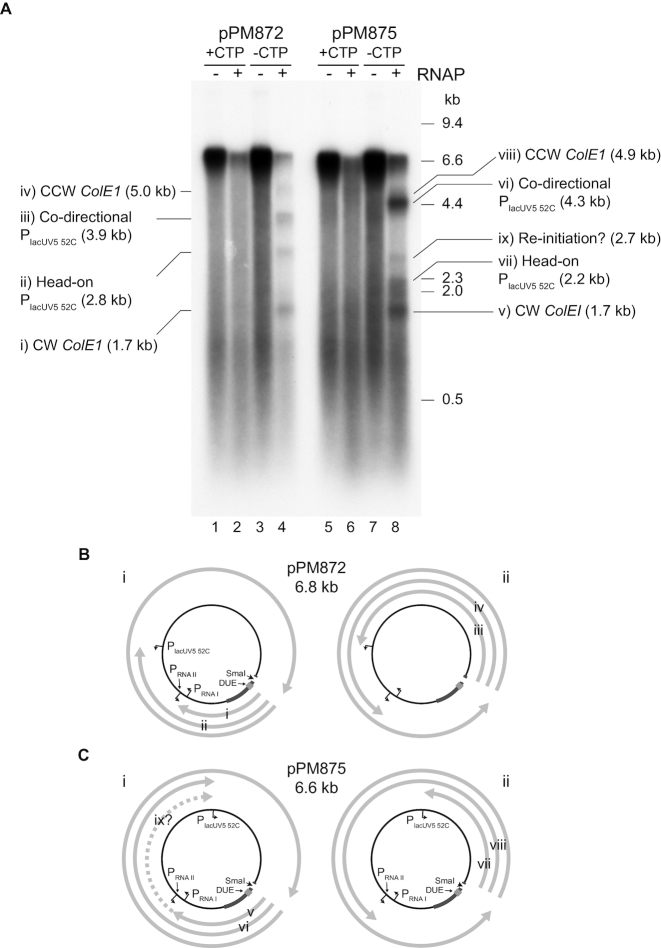
Blockage of the replisome at an engineered transcription elongation complex stall site. (**A**) Denaturing agarose gel of replication products from two plasmids harbouring P_lacUV5 52C_ upstream of either *bla* (pPM872) or *tet* (pPM875). Replication was performed in the presence and absence of RNA polymerase both with and without CTP. (**B** and **C**) Possible truncated leading strand products formed by replisomes originating from *oriC* and moving either clockwise (i) or counter-clockwise (ii) and colliding with RNA polymerases downstream of P_lacUV5 52C_, P_RNAI_ and P_RNAII_. These truncated leading strands are labelled with respect to the gel shown in (A).

The impact of RNA polymerase on *in vitro* replication products using these two templates was analysed in both the presence and the absence of CTP using concentrations of RNA polymerase that had given partial inhibition on pPM854 (Figure 1B, lanes 2 and 6). As with pPM854, addition of RNA polymerase plus CTP resulted in inhibition of full-length leading strands but no prominent truncated leading strands of specific size (Figure 3A, lanes 2 and 6). Omission of CTP led to generation of specific truncated leading strands with both templates (Figure 3A, lanes 4 and 8). These leading strands were sufficiently separated in size to allow correlation with promoter positions (Figure 3B). pPM872 generated four truncated leading strands whose sizes matched those expected for replisomes moving clockwise or counter-clockwise from *oriC* and encountering P_lacUV5 52C_ and the promoters within the *ColE1* plasmid origin of replication (Figure 3A, bands i–iv in lane 4). Note that the RNA I and RNA II promoters within *ColE1* were too close together to analyse the impact of each individual promoter on replication products. The identities of these four bands were confirmed by repeating these blockage experiments on modified versions of pPM872 in which a 0.5 kb DNA fragment was inserted clockwise or counter-clockwise of *oriC* and monitoring the impact of these insertions on truncated leading strand sizes (Supplementary Figure S2). We also extended replication time to test if the replisome eventually overcomes the stalled RNA polymerase barrier (Supplementary Figure S1C). We observed no increase in full-length replication products or decrease in truncated products after 16 min. This reflects the stability of the RNA polymerase block as well as the instability of stalled replisomes. Previous work has established that *in vitro* replisomes retain the ability to resume replication for only 4–6 min (53).

pPM875 generated three major truncated products (Figure 3A, lane 8). As with pPM872, the sizes of truncated leading strand products correlated with predicted sizes of leading strands formed by collisions with P_lacUV5 52C_ and P_RNAI_/P_RNAII_ (Figure 3A, bands v–viii and 3C), given that bands vi and viii were too close in size to resolve. However, there was another band whose size did not correlate with any of the predicted collisions with promoters in this template (Figure 3A, band ix in lane 8). We considered whether this minor 2.7 kb band might be formed by the replisome using mRNA as a primer after colliding with stalled RNA polymerase in the co-directional orientation (15). The only such product that was predicted to be 2.7 kb was one that could be formed by the clockwise moving fork colliding with RNA polymerase downstream of P_RNAI_ and then re-priming using the transcript from P_RNAI_ and continuing around the template to be then blocked by P_lacUV5 52C_ (Figure 3C, band ix). However, the product of transcription from P_RNAI_ (5′-ACAGUAUUUGGUAUCUGCGC-3′) (54) would be predicted to form a stable RNA–DNA hybrid only if transcription proceeded beyond the second nucleotide position encoding cytosine in the absence of CTP. The identity of band ix in Figure 3A was not pursued further. Also note that such a potential re-priming mechanism might also contribute to other leading strand products in these reactions.

### Rep and UvrD promote continuous replication through stalled transcription elongation complexes

**Figure 4.**
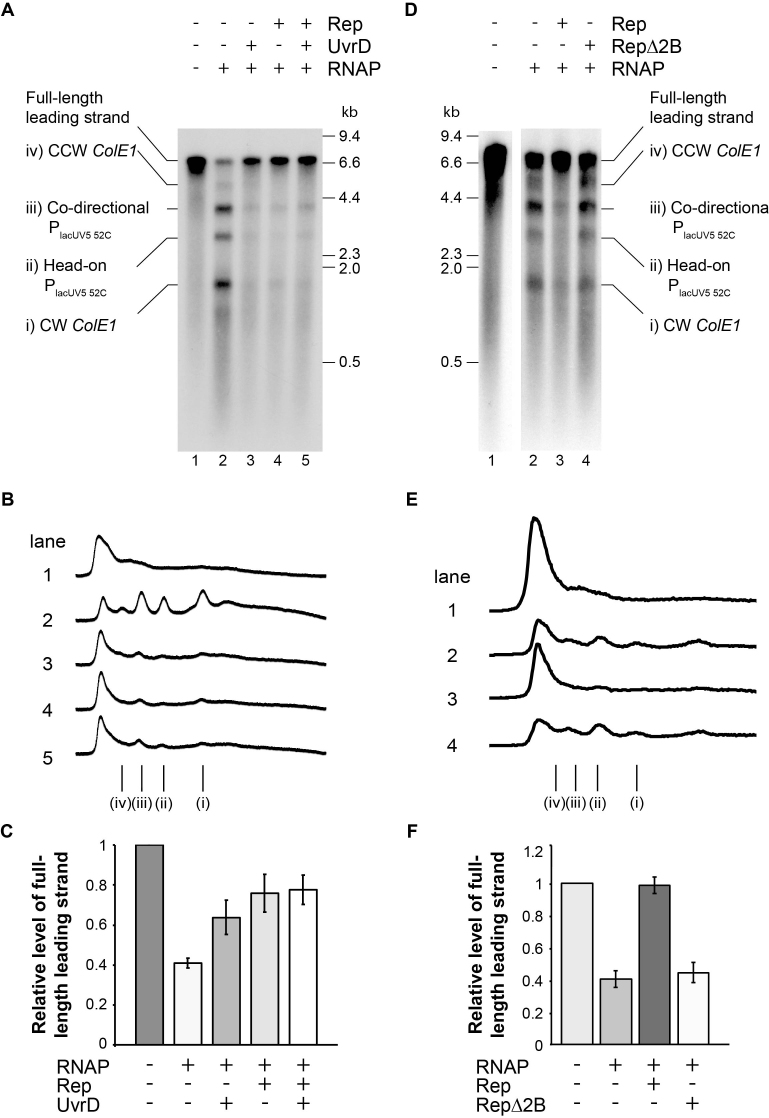
Promotion of replication fork movement through transcribed DNA by Rep and UvrD. (**A**) Denaturing agarose gel of replication products formed from pPM872 in the absence and presence of RNA polymerase, Rep and UvrD as indicated. Truncated leading strand products in lanes 2–5 are labelled according to the diagrams in Figure 3B. (**B**) Phosphorimage profiles of lanes 1–5 in A. Truncated leading strand products are labelled i-iv as in A. (**C**) Relative levels of full-length leading strand formed with respect to the control reaction lacking RNA polymerase, Rep and UvrD (see lane 1 in A). Error bars represent ± one standard error of at least four experiments. (**D–F**) As for (A–C) for RepΔ2B reactions as indicated.

To test whether Rep and UvrD can promote movement of replisomes through stalled RNA polymerase we used pPM872 as a template given the defined truncated leading strand products formed with this plasmid (Figure 3A). Addition of either Rep or UvrD to the replication/transcription reactions with pPM872 resulted in a decrease in all four truncated leading strand products and a concomitant increase in production of full-length leading strands (Figure 4A, compare lanes 3 and 4 with lane 2; Figure 4B and C). Addition of both Rep and UvrD did not lead to any further increase in full- length leading strands (Figure 4C). The decreased intensity of all four truncated leading strand products indicates that both Rep and UvrD promoted replisome movement through stalled RNA polymerase regardless of which promoter the RNA polymerase initiated from and whether the replication/transcription collision was co-directional or head-on (Figure 4B). Furthermore, the increase in full-length leading strand products at the expense of the four truncated leading strands indicates that both Rep and UvrD promote fork movement through transcription complexes without interruption to leading strand synthesis occurring. Thus Rep and UvrD promote fork progression without a need to re-prime leading strand synthesis. This contrasts with the mRNA-dependent re-priming observed after a co-directional collision between a replisome and a stalled transcription elongation complex in the absence of Rep and UvrD (15).

**Figure 5.**
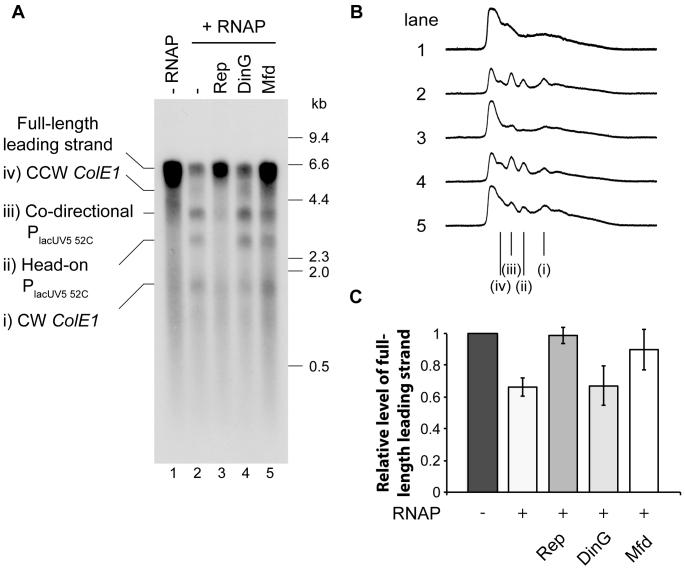
DinG cannot promote replication fork movement through transcribed DNA. (**A**) Denaturing agarose gel of replication products formed from pPM872 in the absence and presence of RNA polymerase plus Rep, DinG or Mfd as indicated. Bands i–iv correspond to those indicated in Figure 3B. (**B**) Phosphorimage profiles of lanes 1–5 in A. Truncated leading strand products i-iv are indicated. (**C**) Relative levels of full-length leading strands formed with respect to the control reaction lacking RNA polymerase (see lane 1 in A). Error bars represent ± one standard error of at least three experiments.

To assess if this activity was dependent on replication we measured RNA polymerase occlusion of the NcoI restriction site (which overlaps the stall site Figure 2B) in the absence of replication forks. Mfd is known to displace stalled RNA polymerase from DNA (8) and reversed the occlusion of the NcoI site by RNA polymerase in the absence of replication (Supplementary Figure S3C and D) as expected. This activity correlated with the ability of Mfd to promote formation of full-length leading strand products in the presence of stalled transcription complexes (Figure 5). In the absence of replication neither Rep nor UvrD had any effect on RNA polymerase occlusion of the NcoI restriction site (Supplementary Figure S3A, B and D). Thus promotion of replication by Rep and UvrD occurs within the context of a replication fork.

Rep is a Superfamily IA helicase and has four domains. Removing the 2B subdomain of Rep promotes hyperactive DNA unwinding *in vitro* (55) but RepΔ2B is deficient in displacing catalytically-inactivated EcoRI endonuclease *in vitro* and cannot complement Δ*rep* Δ*uvrD* lethality (56). Using our system we tested the ability of RepΔ2B to promote movement of replisomes through stalled RNA polymerase (Figure 4D). Addition of RepΔ2B did not reduce the truncated leading strand products and did not increase the production of full-length leading strands. Notably the reaction products with and without RepΔ2B look identical (Figure 4D, compare lanes 2 and 4, Figure 4E and F), indicating that RepΔ2B is completely unable to displace stalled transcription elongation complexes and promote DNA replication.

### DinG has an indirect role in reducing replication and transcription conflicts

DinG helicase has been postulated to act as an accessory replicative helicase in a manner similar to Rep and UvrD (20). However, addition of DinG failed to reduce the formation of truncated leading strand products in the presence of stalled RNA polymerase (Figure 5A–C). DinG activity was confirmed by assessing its ability to unwind a forked substrate with and without a high affinity biotin-streptavidin block (Supplementary Figure S4A–D). The assay was carried out with the radio-label present on either the upper or lower strand and assessed by measuring the presence of streptavidin-less ssDNA (bottom band in S3A and C). These data indicate that DinG removes blocks from the strand it is translocating on in the 5′-3′ direction. This is in contrast to Rep which removes the streptavidin block when translocating in the 3′-5′ direction (44) and RepΔ2B which is extremely inefficient at removing blocks from DNA (56). These data demonstrate that our DinG preparation is active and support the conclusion that DinG cannot act as an accessory replicative helicase to promote replication of transcribed DNA in our *in vitro* assay (see discussion).

**Figure 6.**
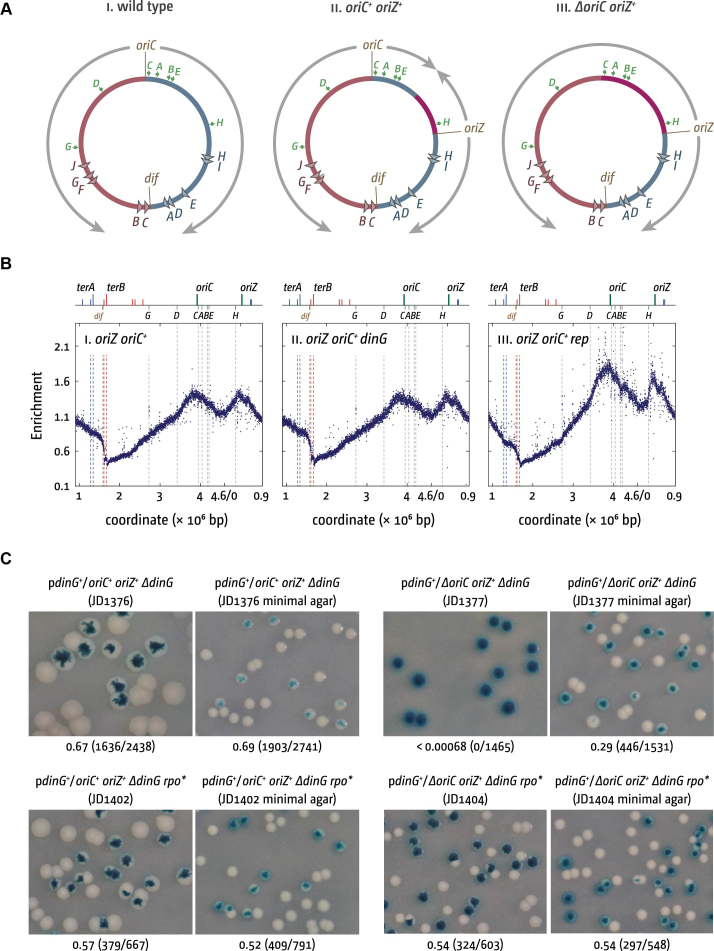
Maintenance of chromosome replication and cell viability of *oriC^+^ oriZ^+^* and *ΔoriC oriZ^+^* cells in the absence of DinG helicase. (**A**) Schematic representation of the replichore arrangement of one or two replication origins in *E. coli*. The origins *oriC* and *oriZ* as well as the *dif* chromosome dimer resolution site are highlighted. Replichores and replication directionality are indicated by grey arrows. *ter* sites are indicated by triangles and identified by their corresponding letter (‘*A*’ indicates the *terA* site). Green arrows represent location and direction of transcription of the 7 *rrn* operons *A–E, G* and *H*. Chromosomal sections in which the direction of DNA replication is artificially inverted because of the presence of an ectopic replication origin are shown in purple. (**B**) Replication profiles of *oriC^+^ oriZ^+^* strains in the absence of either DinG or Rep helicases. The replication profiles are generated by plotting the number of sequence reads (normalized against reads for a stationary phase wild type control) against their chromosomal location. The schematic representation of the *E. coli* chromosome above each panel shows the positions of the two origins, *oriC* and *oriZ*, and *ter* sites (above) as well as the *dif* chromosome dimer resolution site and *rrn* operons *A–E, G* and *H* (below). The strains used were RCe504 (*oriC^+^ oriZ^+^*) and JD1277 (*oriC^+^ oriZ^+^* Δ*dinG*). Data for JD1141 (*oriC^+^ oriZ^+^* Δ*rep*) were re-plotted from Dimude *et al.* (59). All three constructs were sequenced in parallel. (**C**) The plate photographs shown are of synthetic lethality assays, as described in Supplementary Methods. The relevant genotype of the construct used is shown above each photograph, with the strain number in parentheses. The fraction of white colonies is shown below, with the number of white colonies/total colonies analysed in parentheses. The plasmid used was pJD001 (*dinG^+^*) (see Supplementary Information).

To investigate whether DinG promotes replication of transcribed DNA *in vivo*, we used a background in which a second copy of *oriC* (termed *oriZ*) is integrated half-way into the right-hand replichore (45,57). Both origins are simultaneously active (45,57,58). Synthesis initiating at *oriZ* and proceeding counter-clockwise will replicate a stretch of the chromosome that contains the highly-transcribed *rrn* operon *H* in the opposite direction to normal (Figure 6A). *oriC^+^ oriZ^+^* cells grow with few signs of problems, indicating that the full complement of accessory and repair enzymes enable robust progression of replisomes through this area in an atypical direction (45,57).

This changes dramatically in the absence of Rep helicase (59). *oriC^+^ oriZ^+^* Δ*rep* cells could be generated without much difficulty, but the replication profile demonstrates that the vast majority of forks coming from *oriZ* and proceeding against the normal orientation of DNA replication are arrested at *rrnH* (Figure 6Biii) (59). In line with this result we were unable to generate Δ*oriC oriZ^+^* Δ*rep* cells, as the fork proceeding counter-clockwise from *oriZ* will get blocked at *rrnH* or another *rrn* operon in this chromosomal region (Figure 6A), while the second fork traversing in clockwise orientation will be arrested within the termination area by *ter*/Tus fork traps (59). Deletion of the native *oriC* was only possible in Δ*rep* cells if an rpo* RNA polymerase destabilizing point mutation was present (5,60). Replication profiles confirmed that in *oriC^+^ oriZ^+^* Δrep rpo* cells replication can proceed beyond *rrnH* and Δ*oriC oriZ^+^* Δrep rpo* cells grew robustly on both LB broth and minimal salts media (59). These data are in excellent agreement with the Rep *in vitro* data in this study and support the idea that Rep helicase is critical to promote replication of transcribed DNA and highly transcribed areas in particular.

We performed an analogous investigation in cells lacking DinG helicase. In contrast to cells lacking Rep, forks proceed beyond *rrnH* without major issue in *oriC^+^ oriZ^+^* Δ*dinG* cells (Figure 6Bii), in line with our *in vitro* observations. However, by using a synthetic lethality assay where wild type *dinG* is supplied on a mini-F plasmid that can be rapidly lost (46,47), (Supplementary Methods), we were able to demonstrate that despite the ability of forks to move past *rrnH* in Δ*dinG* cells, a Δ*oriC oriZ^+^* Δ*dinG* construct is not viable on LB medium. However growth of white colonies that had lost the covering plasmid was observed on minimal medium (Figure 6C). This lethality was robustly suppressed by a rpo* point mutation, suggesting that it is caused by some form of replication-transcription conflict. Thus, while there is little indication that DinG directly promotes replisome movement through stalled transcription complexes, our results are consistent with a role in promotion of replication of transcribed DNA, as reported (20). However, DinG seems to act via a different and potentially indirect mechanism.

## DISCUSSION

Here, we demonstrate blockage of *E. coli* replisomes by transcription complexes and show that Rep and UvrD can both alleviate this blockage. Rep- and UvrD-directed resolution of conflicts between replication and transcription result in generation of full-length leading strands. Re-priming of leading strand synthesis is therefore not needed for continued fork movement. Removal of elongating transcription complexes by Rep and UvrD to promote replication cannot occur remote from the replication fork, in contrast to Mfd, supporting a model in which these two helicases operate on DNA at the replication fork during collisions (19). *E. coli* therefore possesses two helicases that can operate at the fork in cases when RNAP has not been dissociated from DNA by other means before the replication fork arrives. In contrast, DinG does not promote replication of transcribed DNA in our reconstituted system but our data support the idea that DinG may operate indirectly *in vivo* to reduce conflicts between replication and transcription (20).

The multiple promoters and probable non-specific initiation elsewhere on the DNA complicated our analysis of the impact of specific transcription complexes on replisome movement (Figure 1). RNA polymerase occupancy at P_lacUV5 52C_ is <100% (Figure 2D) and this is also likely to be the case for the *ColE1* promoters. Therefore some forks travelling counter-clockwise will not be blocked at P_lacUV5 52C_ and will go on to be blocked at *ColE1* or *vice versa*. Some replisomes encounter no blocks, leading to synthesis of full-length replication products. The inability to quantify the number of replisomes encountering each block means we could not compare the relative efficiencies of replication blockage by transcription complexes in head-on versus co-directional collisions. We also could not quantify any mRNA-dependent re-priming of leading strand synthesis during co-directional collisions in the absence of Rep and UvrD, although there is some evidence that such re-priming did occur, at least with pPM875 (Figure 3). However, our data show that Rep and UvrD can each promote replisome movement through multiple different types of stalled transcription complex (Figure 4). Such broad activity is essential for reducing replication-transcription conflicts *in vivo*, given the wide variety of transcription complexes that replisomes must encounter inside cells. This ability to promote replisome movement along protein-bound DNA regardless of block identity is also reflected in the ability of Rep and UvrD to also promote replisome movement through non-cleaving EcoRI-DNA complexes *in vitro* (19).

Our data reveal that Rep and UvrD promote the movement of replisomes through transcription complexes without any need to re-prime leading strand synthesis, resulting in the formation of full-length leading strands (Figure 4). Previous work indicated that during co-directional collisions between *E. coli* replisomes and stalled transcription complexes, the replisome can displace RNA polymerase but continued DNA replication depends upon re-priming of leading strand synthesis using the mRNA (15). Such mRNA-dependent re-priming has been suggested to be a potent source of double-stranded DNA breaks since the single-stranded gap in the leading strand caused by re-priming might not be repaired prior to arrival of another replication fork in the next round of chromosome duplication (6). Our data indicate that accessory helicases at the replication fork minimize the need for mRNA-dependent re-priming and any associated dangers of double-strand break formation. The promotion of full-length leading strand synthesis by Rep and UvrD also indicates that re-priming of leading strand synthesis by DnaG, as seen during bypass of lesions in the leading strand template by *E. coli* replisomes (61), is not required. The multiple advantages of accessory helicase-mediated conflict resolution may be why accessory helicases are so prevalent in viruses, bacteria and eukaryotes.

During head-on collisions between replisomes and transcription complexes, re-priming of leading strand synthesis using the mRNA is not possible due to the mRNA being annealed to the lagging strand template. A previous study detected continued extension of the leading strand during a head-on collision with RNA polymerase by displacement of the RNA polymerase from DNA (16). Our data indicate that Rep and UvrD can accelerate the process of resolving head-on collisions. Such acceleration may be critical since paused *E. coli* replisomes have a very limited half life of less than six minutes both *in vitro* and *in vivo* (49,53,62). Indeed, transcription complexes are the primary sources of replication fork pausing in *E. coli* and Rep has been shown to be critical for minimizing the frequency and/or duration of these pauses (29).

Rep rather than UvrD acts as the accessory replicative helicase in wild type *E. coli* due to its ability to interact physically and functionally with the primary replicative helicase DnaB (19,27,29,63). Our RepΔ2B results support the work of Bruning *et al.* (56) and demonstrate that the 2B domain is crucial for Rep-dependent promotion of DNA replication through stalled transcription complexes. Loss of the 2B domain does not impair the DnaB-Rep interaction so localisation of Rep at the replication fork is likely preserved in the mutant (19). This suggests that the 2B domain has a mechanistic role in displacing obstacles to replication.

UvrD is a close homologue of Rep but provides only partial compensation for the absence of Rep function inside cells (19,29,59). The demonstration by this current study that UvrD can promote replication of transcribed DNA *in vitro* lends further support to the view that UvrD can act as an accessory replicative helicase (Figure 4). Given that addition of UvrD to RNA polymerase stalled at the NcoI site in P_lacUV5 52C_ failed to alter the degree of cleavage at this restriction site, our data suggest that the proposed UvrD-induced backtracking of RNA polymerase to facilitate DNA repair (31) is unlikely to be an efficient reaction under these conditions (Supplementary Figure S3D). Indeed, it is difficult to see how UvrD-induced backtracking of RNA polymerase would lead to the facilitation of replisome movement through transcription complexes shown here (Figure 4).

Given that the role of DinG is ambiguous, we combined *in vitro* and *in vivo* work to assess its function. Our data demonstrate that Δ*dinG* cells cannot survive if the chromosome is replicated exclusively from an ectopic origin (Figure 6A). The robust suppression of this effect by an rpo* point mutation which destabilizes RNA polymerase, as well as growth in minimal medium (Figure 6), supports the idea that DinG underpins replication of highly-transcribed areas in *E. coli* (20,64). However, our *in vitro* data demonstrate that DinG does not directly promote replisome movement through stalled transcription complexes (Figure 5) and the *in vivo* replication profiles of *oriC^+^ oriZ^+^* Δ*dinG* cells do not show any perturbance of DNA replication at *rrnH* (Figure 6Bii), in stark contrast to cells lacking Rep (59). The mean replication fork speed is significantly reduced in Δ*rep* cells (27,65), which is reflected in the increased origin/terminus ratio observed in the replication profiles of Δ*rep* single mutants as well as *oriC^+^ oriZ^+^* Δ*rep* cells (Figure 6Biii) (59). No such effect is seen in *oriC^+^ oriZ^+^* Δ*dinG* cells (Figure 6Bii). Thus, the replication profiles do not provide any evidence that DinG is involved directly in resolving replication-transcription encounters at highly transcribed regions (Figure 6Bii). Thus, our *in vitro* and *in vivo* data suggest that any DinG-promoted replication of transcribed DNA is therefore likely to be indirect, possibly via its ability to unwind RNA:DNA hybrids (38). Indeed, the 5′-3′ polarity of DinG translocation (37) means that this Superfamily 2 helicase cannot function in the same manner as Rep or UvrD. Rep and UvrD likely function by binding and translocating along the leading strand template. Their 3′-5′ polarity means that they would move ahead of the advancing replisome towards any potential nucleoprotein barriers (19). We show here that DinG only removes blocks from the strand it is translocating on (Supplemental Figure S4). The 5′-3′ polarity of DinG prevents DinG from moving ahead of the replisome along the leading strand template (23). DinG could conceivably translocate 5′-3′ along the lagging strand template towards potential nucleoprotein blocks. However, it is difficult to envisage sufficient single-stranded DNA being exposed on the lagging strand template given that the primary replicative helicase DnaB would also be translocating forward on the lagging strand template. Further characterization of the indirect role played by DinG in reducing replication-transcription conflicts is a challenge for future work.

Our direct demonstration of the ability of accessory replicative helicases to promote replication of transcribed DNA underlines the importance of RNA polymerases as physical barriers to replication fork movement. The experimental system established here can also be adapted for further analysis of the molecular mechanisms underlying these conflicts. The essentiality of accessory replicative helicases and their function in disrupting potential nucleoprotein barriers ahead of replication forks (19–21) clearly indicate that RNA polymerases on template DNA are important physical barriers to DNA replication regardless of the genome instability problems posed by R-loops (10,11).

## Supplementary Material

gkz170_Supplemental_File

## References

[1] Brewer B.J. When polymerases collide: replication and the transcriptional organization of the *E. coli* chromosome. Cell. 1988; 53:679–686. 3286014 10.1016/0092-8674(88)90086-4

[2] Thomas B.J., Rothstein R. Elevated recombination rates in transcriptionally active DNA. Cell. 1989; 56:619–630. 2645056 10.1016/0092-8674(89)90584-9

[3] Vilette D., Uzest M., Ehrlich S.D., Michel B. DNA transcription and repressor binding affect deletion formation in *Escherichia coli* plasmids. EMBO J. 1992; 11:3629–3634. 1396563 10.1002/j.1460-2075.1992.tb05447.x PMC556822

[4] Gaillard H., Aguilera A. Transcription as a threat to genome integrity. Annu. Rev. Biochem. 2016; 85:291–317. 27023844 10.1146/annurev-biochem-060815-014908

[5] Trautinger B.W., Jaktaji R.P., Rusakova E., Lloyd R.G. RNA polymerase modulators and DNA repair activities resolve conflicts between DNA replication and transcription. Mol. Cell. 2005; 19:247–258. 16039593 10.1016/j.molcel.2005.06.004

[6] Dutta D., Shatalin K., Epshtein V., Gottesman M.E., Nudler E. Linking RNA polymerase backtracking to genome instability in *E. coli*. Cell. 2011; 146:533–543. 21854980 10.1016/j.cell.2011.07.034 PMC3160732

[7] Selby C.P., Sancar A. Molecular mechanism of transcription-repair coupling. Science. 1993; 260:53–58. 8465200 10.1126/science.8465200

[8] Park J.S., Marr M.T., Roberts J.W. *E. coli* transcription repair coupling factor (mfd protein) rescues arrested complexes by promoting forward translocation. Cell. 2002; 109:757–767. 12086674 10.1016/s0092-8674(02)00769-9

[9] Huertas P., Aguilera A. Cotranscriptionally formed DNA:RNA hybrids mediate transcription elongation impairment and transcription-associated recombination. Mol. Cell. 2003; 12:711–721. 14527416 10.1016/j.molcel.2003.08.010

[10] Lang K.S., Hall A.N., Merrikh C.N., Ragheb M., Tabakh H., Pollock A.J., Woodward J.J., Dreifus J.E., Merrikh H. Replication-Transcription conflicts generate R-Loops that orchestrate bacterial stress survival and pathogenesis. Cell. 2017; 170:787–799. 28802046 10.1016/j.cell.2017.07.044 PMC5630229

[11] Hamperl S., Bocek M.J., Saldivar J.C., Swigut T., Cimprich K.A. Transcription-Replication conflict orientation modulates R-Loop levels and activates distinct DNA damage responses. Cell. 2017; 170:774–786. 28802045 10.1016/j.cell.2017.07.043 PMC5570545

[12] Brambati A., Colosio A., Zardoni L., Galanti L., Liberi G. Replication and transcription on a collision course: eukaryotic regulation mechanisms and implications for DNA stability. Front. Genet. 2015; 6:166. 25972894 10.3389/fgene.2015.00166 PMC4412130

[13] Mirkin E.V., Mirkin S.M. Mechanisms of transcription-replication collisions in bacteria. Mol. Cell. Biol. 2005; 25:888–895. 15657418 10.1128/MCB.25.3.888-895.2005 PMC544003

[14] Merrikh H., Machon C., Grainger W.H., Grossman A.D., Soultanas P. Co-directional replication-transcription conflicts lead to replication restart. Nature. 2011; 470:554–557. 21350489 10.1038/nature09758 PMC3059490

[15] Pomerantz R.T., O¢Donnell M. The replisome uses mRNA as a primer after colliding with RNA polymerase. Nature. 2008; 456:762–766. 19020502 10.1038/nature07527 PMC2605185

[16] Pomerantz R.T., O¢Donnell M. Direct restart of a replication fork stalled by a head-on RNA polymerase. Science. 2010; 327:590–592. 20110508 10.1126/science.1179595 PMC2861996

[17] McGlynn P., Savery N.J., Dillingham M.S. The conflict between DNA replication and transcription. Mol. Microbiol. 2012; 85:12–20. 22607628 10.1111/j.1365-2958.2012.08102.x

[18] Ivessa A.S., Lenzmeier B.A., Bessler J.B., Goudsouzian L.K., Schnakenberg S.L., Zakian V.A. The *Saccharomyces cerevisiae* helicase Rrm3p facilitates replication past nonhistone protein-DNA complexes. Mol. Cell. 2003; 12:1525–1536. 14690605 10.1016/s1097-2765(03)00456-8

[19] Guy C.P., Atkinson J., Gupta M.K., Mahdi A.A., Gwynn E.J., Rudolph C.J., Moon P.B., van Knippenberg I.C., Cadman C.J., Dillingham M.S. et al. Rep provides a second motor at the replisome to promote duplication of Protein-Bound DNA. Mol. Cell. 2009; 36:654–666. 19941825 10.1016/j.molcel.2009.11.009 PMC2807033

[20] Boubakri H., de Septenville A.L., Viguera E., Michel B. The helicases DinG, Rep and UvrD cooperate to promote replication across transcription units *in vivo*. EMBO J. 2010; 29:145–157. 19851282 10.1038/emboj.2009.308 PMC2770101

[21] Sabouri N., McDonald K.R., Webb C.J., Cristea I.M., Zakian V.A. DNA replication through hard-to-replicate sites, including both highly transcribed RNA Pol II and Pol III genes, requires the *S. pombe* Pfh1 helicase. Genes Dev. 2012; 26:581–593. 22426534 10.1101/gad.184697.111 PMC3315119

[22] Steinacher R., Osman F., Dalgaard J.Z., Lorenz A., Whitby M.C. The DNA helicase Pfh1 promotes fork merging at replication termination sites to ensure genome stability. Genes Dev. 2012; 26:594–602. 22426535 10.1101/gad.184663.111 PMC3315120

[23] Bruning J.G., Howard J.L., McGlynn P. Accessory replicative helicases and the replication of Protein-Bound DNA. J. Mol. Biol. 2014; 426:3917–3928. 25308339 10.1016/j.jmb.2014.10.001

[24] Yarranton G.T., Gefter M.L. Enzyme-catalyzed DNA unwinding: studies on *Escherichia coli* rep protein. Proc. Natl. Acad. Sci. U.S.A. 1979; 76:1658–1662. 221901 10.1073/pnas.76.4.1658 PMC383449

[25] Matson S.W. *Escherichia coli* helicase II (*urvD* gene product) translocates unidirectionally in a 3′ to 5′ direction. J. Biol. Chem. 1986; 261:10169–10175. 2942537

[26] Uzest M., Ehrlich S.D., Michel B. Lethality of *rep recB* and *rep recC* double mutants of *Escherichia coli*. Mol. Microbiol. 1995; 17:1177–1188. 8594336 10.1111/j.1365-2958.1995.mmi_17061177.x

[27] Atkinson J., Gupta M.K., Rudolph C.J., Bell H., Lloyd R.G., McGlynn P. Localization of an accessory helicase at the replisome is critical in sustaining efficient genome duplication. Nucleic Acids Res. 2011; 39:949–957. 20923786 10.1093/nar/gkq889 PMC3035471

[28] Syeda A.H., Atkinson J., Lloyd R.G., McGlynn P. The balance between recombination enzymes and accessory replicative helicases in facilitating genome duplication. Genes (Basel). 2016; 7:42. 10.3390/genes7080042 PMC4999830 27483323

[29] Gupta M.K., Guy C.P., Yeeles J.T., Atkinson J., Bell H., Lloyd R.G., Marians K.J., McGlynn P. Protein-DNA complexes are the primary sources of replication fork pausing in *Escherichia coli*. Proc. Natl. Acad. Sci. U.S.A. 2013; 110:7252–7257. 23589869 10.1073/pnas.1303890110 PMC3645559

[30] Gwynn E.J., Smith A.J., Guy C.P., Savery N.J., McGlynn P., Dillingham M.S. The conserved C-Terminus of the PcrA/UvrD helicase interacts directly with RNA polymerase. PLoS One. 2013; 8:e78141. 24147116 10.1371/journal.pone.0078141 PMC3797733

[31] Epshtein V., Kamarthapu V., McGary K., Svetlov V., Ueberheide B., Proshkin S., Mironov A., Nudler E. UvrD facilitates DNA repair by pulling RNA polymerase backwards. Nature. 2014; 505:372–377. 24402227 10.1038/nature12928 PMC4471481

[32] Sanders K., Lin C.L., Smith A.J., Cronin N., Fisher G., Eftychidis V., McGlynn P., Savery N.J., Wigley D.B., Dillingham M.S. The structure and function of an RNA polymerase interaction domain in the PcrA/UvrD helicase. Nucleic Acids Res. 2017; 45:3875–3887. 28160601 10.1093/nar/gkx074 PMC5397179

[33] Adebali O., Chiou Y.Y., Hu J., Sancar A., Selby C.P. Genome-wide transcription-coupled repair in *Escherichia coli* is mediated by the Mfd translocase. Proc. Natl. Acad. Sci. U.S.A. 2017; 114:E2116–E2125. 28167766 10.1073/pnas.1700230114 PMC5358382

[34] Adebali O., Sancar A., Selby C.P. Mfd translocase is necessary and sufficient for transcription-coupled repair in Escherichia coli. J. Biol. Chem. 2017; 292:18386–18391. 28986449 10.1074/jbc.C117.818807 PMC5682952

[35] Fan J., Leroux-Coyau M., Savery N.J., Strick T.R. Reconstruction of bacterial transcription-coupled repair at single-molecule resolution. Nature. 2016; 536:234–237. 27487215 10.1038/nature19080

[36] Merrikh C.N., Brewer B.J., Merrikh H. The *B. subtilis* Accessory Helicase PcrA Facilitates DNA Replication through Transcription Units. PLoS Genet. 2015; 11:e1005289. 26070154 10.1371/journal.pgen.1005289 PMC4466434

[37] Voloshin O.N., Vanevski F., Khil P.P., Camerini-Otero R.D. Characterization of the DNA damage-inducible helicase DinG from *Escherichia coli*. J. Biol. Chem. 2003; 278:28284–28293. 12748189 10.1074/jbc.M301188200

[38] Voloshin O.N., Camerini-Otero R.D. The DinG protein from *Escherichia coli* is a structure-specific helicase. J. Biol. Chem. 2007; 282:18437–18447. 17416902 10.1074/jbc.M700376200

[39] Hiasa H., Marians K.J. Tus prevents overreplication of *oriC* plasmid DNA. J. Biol. Chem. 1994; 269:26959–26968. 7929435

[40] Deaconescu A.M., Chambers A.L., Smith A.J., Nickels B.E., Hochschild A., Savery N.J., Darst S.A. Structural basis for bacterial transcription-coupled DNA repair. Cell. 2006; 124:507–520. 16469698 10.1016/j.cell.2005.11.045

[41] Mayer M.P. A new set of useful cloning and expression vectors derived from pBlueScript. Gene. 1995; 163:41–46. 7557476 10.1016/0378-1119(95)00389-n

[42] Atkinson J., Guy C.P., Cadman C.J., Moolenaar G.F., Goosen N., McGlynn P. Stimulation of UvrD helicase by UvrAB. J. Biol. Chem. 2009; 284:9612–9623. 19208629 10.1074/jbc.M808030200 PMC2666613

[43] Manelyte L., Kim Y.I., Smith A.J., Smith R.M., Savery N.J. Regulation and rate enhancement during transcription-coupled DNA repair. Mol. Cell. 2010; 40:714–724. 21145481 10.1016/j.molcel.2010.11.012 PMC3025350

[44] Bruning J.G., Howard J.A., McGlynn P. Use of streptavidin bound to biotinylated DNA structures as model substrates for analysis of nucleoprotein complex disruption by helicases. Methods. 2016; 108:48–55. 27017910 10.1016/j.ymeth.2016.03.017

[45] Ivanova D., Taylor T., Smith S.L., Dimude J.U., Upton A.L., Mehrjouy M.M., Skovgaard O., Sherratt D.J., Retkute R., Rudolph C.J. Shaping the landscape of the *Escherichia coli* chromosome: replication-transcription encounters in cells with an ectopic replication origin. Nucleic Acids Res. 2015; 43:7865–7877. 26160884 10.1093/nar/gkv704 PMC4652752

[46] Bernhardt T.G., de Boer P.A. Screening for synthetic lethal mutants in *Escherichia coli* and identification of EnvC (YibP) as a periplasmic septal ring factor with murein hydrolase activity. Mol. Microbiol. 2004; 52:1255–1269. 15165230 10.1111/j.1365-2958.2004.04063.x PMC4428336

[47] Mahdi A.A., Buckman C., Harris L., Lloyd R.G. Rep and PriA helicase activities prevent RecA from provoking unnecessary recombination during replication fork repair. Genes Dev. 2006; 20:2135–2147. 16882986 10.1101/gad.382306 PMC1536063

[48] Smelkova N., Marians K.J. Timely release of both replication forks from *oriC* requires modulation of origin topology. J. Biol. Chem. 2001; 276:39186–39191. 11504719 10.1074/jbc.M104411200

[49] Marians K.J., Hiasa H., Kim D.R., McHenry C.S. Role of the core DNA polymerase III subunits at the replication fork. α is the only subunit required for processive replication. J. Biol. Chem. 1998; 273:2452–2457. 9442096 10.1074/jbc.273.4.2452

[50] McMacken R., Kornberg A. A multienzyme system for priming the replication of fX174 viral DNA. J. Biol. Chem. 1978; 253:3313–3319. 346590

[51] Hsu L.M. Monitoring abortive initiation. Methods. 2009; 47:25–36. 18948204 10.1016/j.ymeth.2008.10.010 PMC2647590

[52] Komissarova N., Kashlev M. Transcriptional arrest: *Escherichia coli* RNA polymerase translocates backward, leaving the 3′ end of the RNA intact and extruded. Proc. Natl. Acad. Sci. U.S.A. 1997; 94:1755–1760. 9050851 10.1073/pnas.94.5.1755 PMC19989

[53] McGlynn P., Guy C.P. Replication forks blocked by protein-DNA complexes have limited stability *in vitro*. J. Mol. Biol. 2008; 381:249–255. 18602646 10.1016/j.jmb.2008.05.053

[54] Morita M., Oka A. Structure of a transcriptional unit on Colicine-1 plasmid. Eur. J. Biochem. 1979; 97:435–443. 380993 10.1111/j.1432-1033.1979.tb13131.x

[55] Brendza K.M., Cheng W., Fischer C.J., Chesnik M.A., Niedziela-Majka A., Lohman T.M. Autoinhibition of *Escherichia coli* Rep monomer helicase activity by its 2B subdomain. Proc. Natl. Acad. Sci. U.S.A. 2005; 102:10076–10081. 16009938 10.1073/pnas.0502886102 PMC1177377

[56] Bruning J.G., Howard J.A.L., Myka K.K., Dillingham M.S., McGlynn P. The 2B subdomain of Rep helicase links translocation along DNA with protein displacement. Nucleic Acids Res. 2018; 46:8917–8925. 30060236 10.1093/nar/gky673 PMC6158625

[57] Wang X., Lesterlin C., Reyes-Lamothe R., Ball G., Sherratt D.J. Replication and segregation of an *Escherichia coli* chromosome with two replication origins. Proc. Natl. Acad. Sci. U.S.A. 2011; 108:E243–E250. 21670292 10.1073/pnas.1100874108 PMC3127894

[58] Dimude J.U., Stein M., Andrzejewska E.E., Khalifa M.S., Gajdosova A., Retkute R., Skovgaard O., Rudolph C.J. Origins left, right, and Centre: Increasing the number of initiation sites in the escherichia coli chromosome. Genes (Basel). 2018; 9:376. 10.3390/genes9080376 PMC6116050 30060465

[59] Dimude J.U., Midgley-Smith S.L., Rudolph C.J. Replication-transcription conflicts trigger extensive DNA degradation in Escherichia coli cells lacking RecBCD. DNA Repair (Amst.). 2018; 70:37–48. 30145455 10.1016/j.dnarep.2018.08.002

[60] Trautinger B.W., Lloyd R.G. Modulation of DNA repair by mutations flanking the DNA channel through RNA polymerase. EMBO J. 2002; 21:6944–6953. 12486015 10.1093/emboj/cdf654 PMC139083

[61] Yeeles J.T., Marians K.J. The *Escherichia coli* replisome is inherently DNA damage tolerant. Science. 2011; 334:235–238. 21998391 10.1126/science.1209111 PMC3593629

[62] Mettrick K.A., Grainge I. Stability of blocked replication forks *in vivo*. Nucleic Acids Res. 2015; 44:657–668. 26490956 10.1093/nar/gkv1079 PMC4737137

[63] Atkinson J., Gupta M.K., McGlynn P. Interaction of Rep and DnaB on DNA. Nucleic Acids Res. 2011; 39:1351–1359. 20959294 10.1093/nar/gkq975 PMC3045612

[64] Baharoglu Z., Lestini R., Duigou S., Michel B. RNA polymerase mutations that facilitate replication progression in the *rep uvrD recF* mutant lacking two accessory replicative helicases. Mol. Microbiol. 2010; 77:324–336. 20497334 10.1111/j.1365-2958.2010.07208.x PMC2936116

[65] Lane H.E., Denhardt D.T. The rep mutation. IV. Slower movement of replication forks in *Escherichia coli rep* strains. J. Mol. Biol. 1975; 97:99–112. 1100854 10.1016/s0022-2836(75)80025-8

