# Supplementary material for: Direct removal of RNA polymerase barriers to replication by accessory replicative helicases
Source: Nucleic Acids Res. 2019 Mar 14;47(10):5100–13. doi: 10.1093/nar/gkz170 (PMC6547429; doi:10.1093/nar/gkz170)
Supplement: gkz170_Supplemental_File [file gkz170_supplemental_file.pdf]

Supplementary Information

Supplementary Methods

Helicase assay

Unwinding of streptavidin-bound forks was assayed using a substrate made by annealing oligonucleotides PM187B20 and PM188B34. Reactions were performed in final volumes of 10 µl in 40 mM HEPES (pH 8); 10 mM DTT; 10 mM magnesium acetate; 2 mM ATP; 0.1 mg/ml BSA and 1 nM forked DNA substrate. Reactions were carried out as described in (1). Briefly, the reaction mixture was pre-incubated at 37°C for five minutes +/- 1 µM streptavidin (Sigma-Aldrich), then histidine-tagged DinG and biotin (Sigma-Aldrich) to 100 µM (acting as a trap for free streptavidin) were added and incubation continued at 37°C for 10 minutes. Reactions were stopped with 2.5 µl of 2.5% SDS, 200 mM EDTA and 10 mg ml^{-1} of proteinase K and analysed by non-denaturing gel electrophoresis on 10% polyacrylamide gels. The quantification of the unwinding and displacement of streptavidin from the fork was carried out as described (1).

Marker frequency analysis by deep sequencing

Marker frequency analysis by Deep Sequencing was performed as described previously (2-4) with only minor modifications. Samples from cultures of a strain grown over night in LB broth were diluted 100-fold in fresh LB broth and incubated with vigorous aeration until an A_{600} reached 0.48 at 37°C to ensure they were in exponential growth conditions. Cultures were then diluted a second time 100-fold in pre-warmed fresh broth and grown again until an A_{600} of 0.48 was reached. Samples from these exponential phase cultures were flash-frozen in liquid nitrogen at this point for subsequent DNA extraction. For wild type, incubation of the remaining culture was continued until several hours after the culture had saturated and showed no further increase in the A_{600}. A further sample (stationary phase) was frozen at this point. DNA was then extracted using the GenElute Bacterial Genomic DNA Kit (Sigma-Aldrich). Marker frequency analysis was performed using Illumina HiSeq 2500 sequencing (fast run) to measure sequence copy number. FastQC was used for a basic metric of quality control in the raw data. Bowtie2 was used to align the sequence reads to the reference. Samtools was used to calculate the enrichment of uniquely mapped sequence tags in 1 kb windows.

For presentation of the data as a marker frequency replication profile the raw read counts for each construct were divided by the average of all read counts across the entire genome to correct for the somewhat different absolute numbers of aligned

reads in the various samples. The normalised read count values for each exponentially growing sample were then divided by the corresponding normalised read count value from a stationary (non-replicating) sample. This division "cleans" the raw data significantly, because data points which are outliers caused by technical aspects (precise sequence environment interfering with library preparation or similar issues) will be similarly distorted both in the exponential and the stationary samples.

Synthetic lethality assay

The synthetic lethality assay was performed as described (5,6). In essence, a wild type copy of a gene of interest (*dinG*) under its native promoter was cloned into the ApaI site of pRC7 by introducing ApaI site at both ends of the PCR product, giving plasmid pJD001. pRC7 is a lac^+ mini-F plasmid that is rapidly lost. The resulting p$dinG^+$ construct was then used to cover the deletion of the chromosomal *dinG* deletion in a Δlac^- background. Additional mutations can then be introduced to test for synthetic lethality with the deleted allele. If synthetically lethal, cells that lose the plasmid will fail to grow and only lac^+ colonies formed by cells retaining the plasmid will be observed. When viability is reduced but not eliminated, colonies formed by cells retaining the plasmid are noticeably larger than white colonies formed by plasmid-free cells. Cultures of strains carrying the relevant pRC7 derivatives were grown overnight in LB broth containing 100 µg/ml ampicillin to maintain plasmid selection, diluted 100-fold in LB broth and grown without ampicillin selection to an A_{600} of 0.4 before spreading dilutions on LB agar or M9 glucose minimal salts agar supplemented with 120 µg/ml X-gal and 1 mM IPTG. Plates were photographed and scored after 48 h (LB agar) or 72 h (M9 agar) at 37°C. At least two independent experiments were performed for each construct investigated.

Supplementary Figures

Supplementary Figure 1. RNA polymerase stalled at $P_{lacUV5\ 52C}$ is a stable block. (**A**) RNA polymerase occludes the NcoI restriction site within $P_{lacUV5\ 52C}$. RNA polymerase was added to pPM872 with ATP, GTP and UTP but in the absence of CTP. At time zero a sample was removed and digested with NcoI (lane 1), further samples for NcoI digestion were removed at 10, 30 and 60 minute time points (lanes 2-4). The formation of linearised plasmid indicated occlusion of the NcoI site by stalled RNA polymerase. Stalled RNA polymerase is still present after 60 minutes and therefore this block is chronic. (**B**) Relative levels of linearised plasmid compared to completely cut plasmid with respect to the zero time point. Error bars

represent +/- one standard error of four experiments. (**C**) Denaturing agarose gel showing a time course of replication products when RNA polymerase is stalled on pPM872 in the absence of CTP. Following addition of SmaI and [α^{32}P] dCTP, the reaction was incubated at 37°C for 1 minute and a 15 µl sample was removed and terminated. The remaining reaction was quenched with excess dCTP (4 mM final concentration) to minimise further radionucleotide incorporation and incubation continued at 37°C. Samples were removed at the times indicated and terminated.

Supplementary Figure 2. The identities of the truncated leading strand products formed in the presence of RNA polymerase on pPM872 (**A**) were probed using by insertion of a 0.5 kb DNA fragment either clockwise (**B**) or counter-clockwise (**C**) from *oriC*. The putative identification of truncated leading strand products i-iv in Figure 3 using pPM872 as template predict that these inserts should result in increases in size of bands i and ii with pMH049 and bands iii and iv with pMH033, in addition to an increased full-length leading strand product. These predicted size increases are shown in (**D**). (**E**) Denaturing agarose gel of replication products formed from pPM872, pMH049 and pMH033 in the presence of RNA polymerase. Bands i-iv indicate the truncated leading strand products formed with pPM872 in lane 2. As can be seen in lanes 3 and 4, bands i and ii increase in size with pMH049 as template whilst bands iii and iv increase in size with pMH033. Note that the new 3.4 kb leading strand formed with pMH049 migrates just below the 3.9 kb product. These altered leading strand patterns support the proposed identities of the truncated leading strands formed by pPM872 in the presence of RNA polymerase.

Supplementary Figure 3. The ability of (**A**) Rep and (**B**) UvrD to prevent occlusion by RNA polymerase of the NcoI restriction site within $P_{lacUV5\ 52C}$ in the absence of a replication fork was tested. RNA polymerase was added to pPM872 with ATP, GTP and UTP but in the absence of CTP. Rep or UvrD were added as indicated. At time zero a sample was removed and digested with NcoI (lane 1). Samples for NcoI digestion were removed 1, 2 and 4 minutes (lanes 2-4) after addition of CTP. The formation of linearised plasmid indicated occlusion of the NcoI site by stalled RNA polymerase. Neither Rep nor UvrD altered the fraction of linearised plasmid when CTP was absent (lanes 1-4). In contrast, when CTP was added alongside either helicase then the linearised plasmid band disappeared, indicating that stalled RNA polymerase was no longer inhibiting cleavage of the NcoI site within $P_{lacUV5\ 52C}$ (lanes 5-8). These data demonstrate that neither Rep nor UvrD could displace RNA

polymerase from this NcoI site in the absence of a replication fork. As a control we utilised the known ability of Mfd to displace stalled RNA polymerase from DNA (7). (**C**) The experiments in A and B were repeated in the same manner but using Mfd rather than Rep or UvrD. Production of linearised plasmid by NcoI cleavage was abolished upon addition of Mfd regardless of the absence or presence of CTP (lanes 1-4 and 5-8 respectively). Thus Mfd, as expected, could displace RNA polymerase from this NcoI site in the absence of a replication fork, in contrast to either Rep or UvrD. (**D**) Relative levels of linearised plasmid compared to completely cut plasmid with respect to the zero point. Error bars represent +/- one standard error of three experiments.

Supplementary Figure 4. DinG can remove streptavidin-biotin blocks on fork substrates. (**A**) and (**C**) Native polyacrylamide gel of DinG-catalysed unwinding of forked DNA substrate bearing biotin groups on both strands within the duplex portion of the fork. The radio-label is on the upper (**A**) and lower (**C**) strand. Lanes 1–4 contain markers indicating the positions of single-stranded and double-stranded DNA without and with bound streptavidin as indicated. Lanes 5–12 contain the products of unwinding of the forked DNA substrate in the absence and presence of streptavidin. DinG was present at 1, 10, 50 and 100 nM final concentration. (**B**) and (**D**) Quantification of biotinylated fork unwinding where the label is on the upper and lower strand respectively. Error bars represent +/- one standard error of two and four experiments respectively. White squares represent reactions without streptavidin and black squares represent reactions with streptavidin.

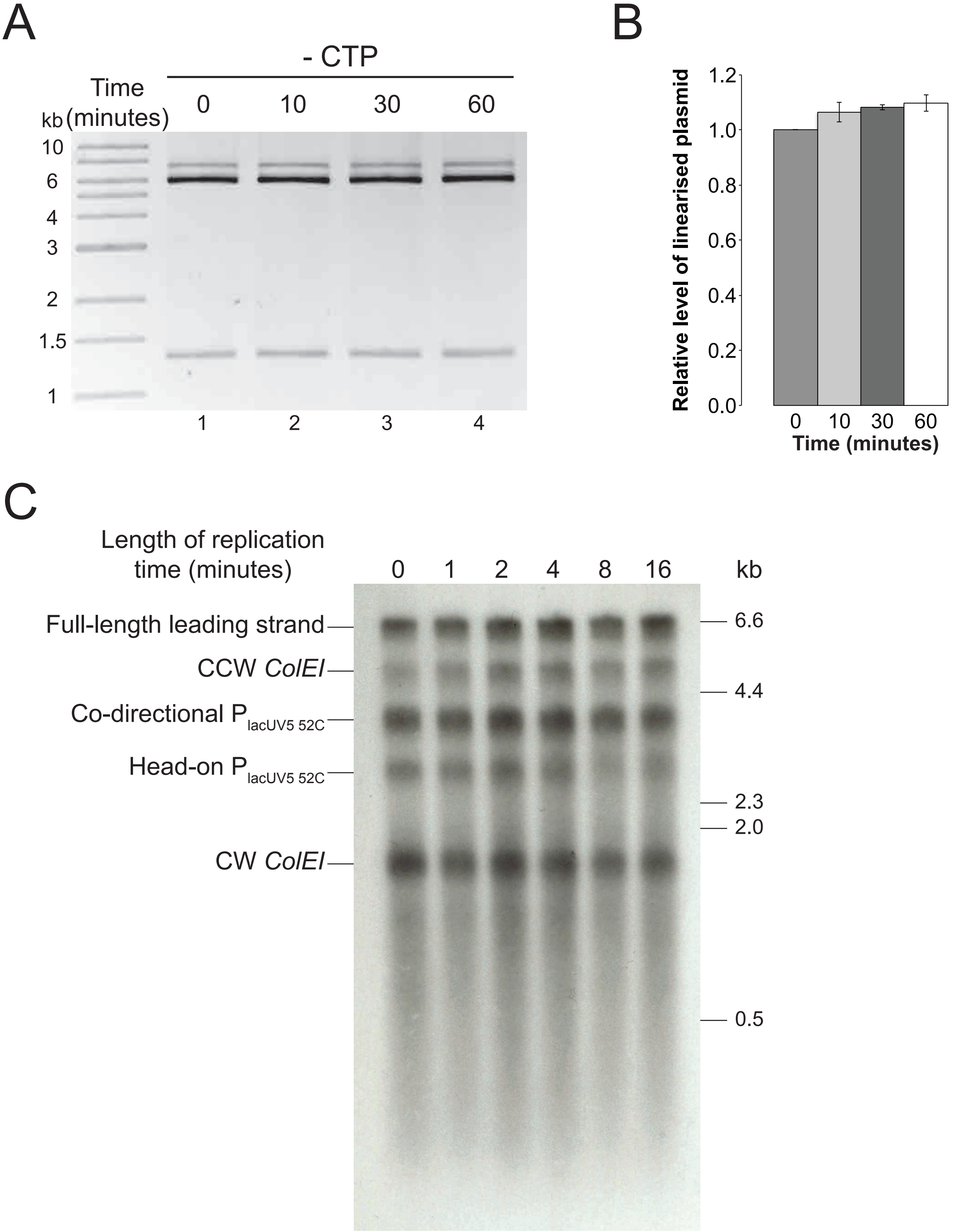

Supplementary Figure 1

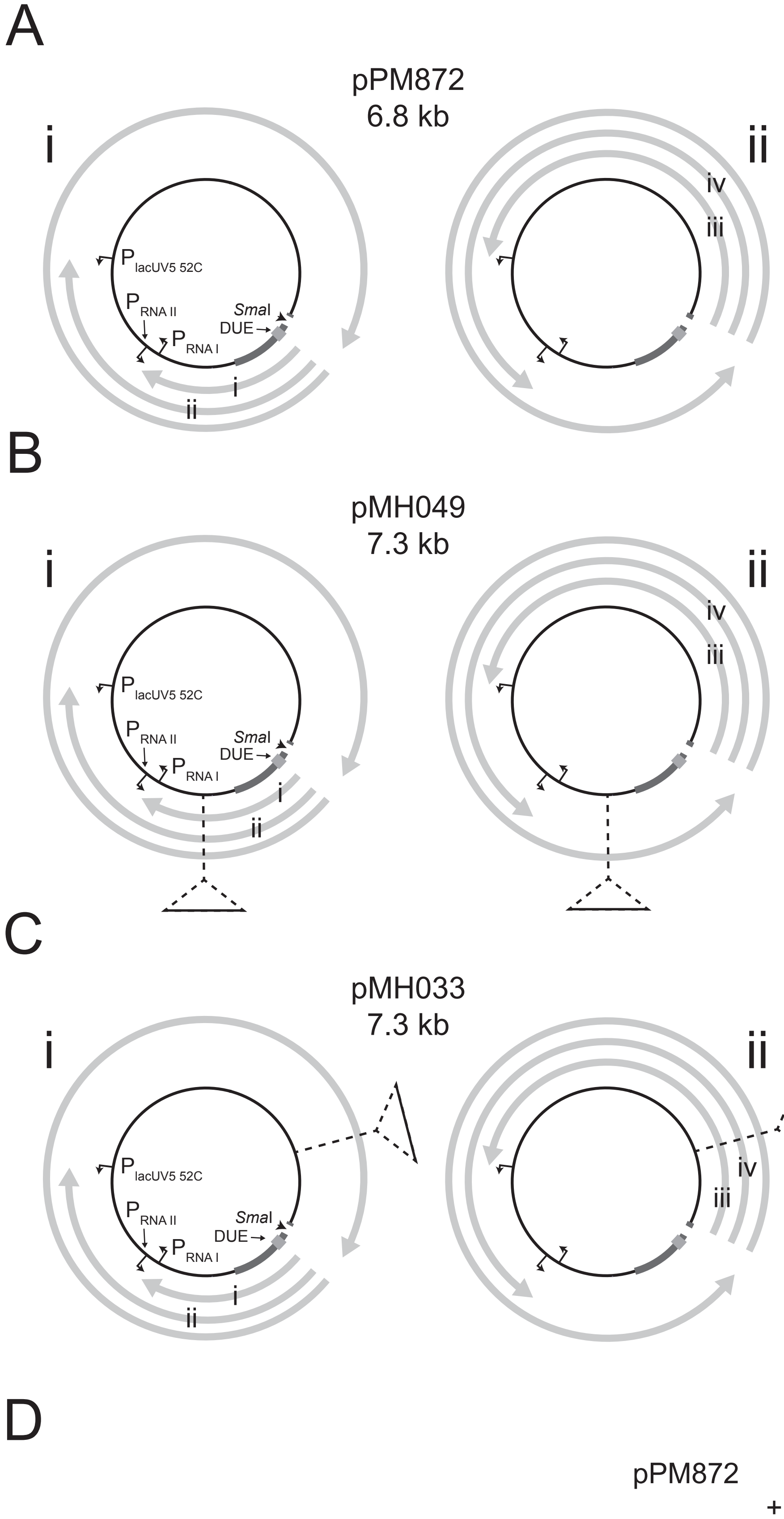

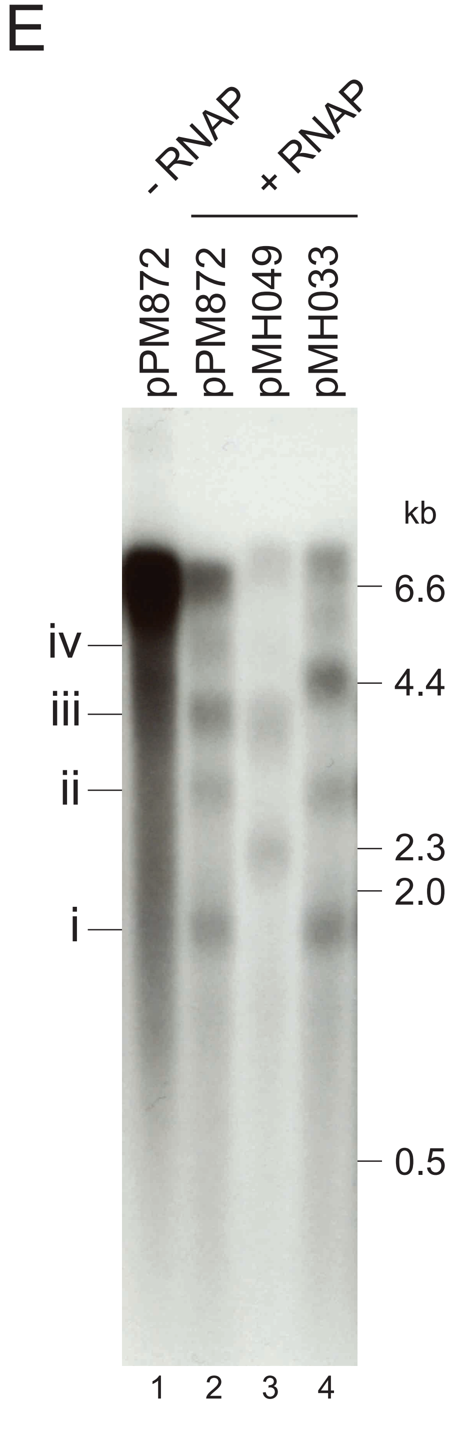

D

	pPM872	pMH049 + 0.5 kb CW	pMH033 + 0.5 kb CCW
i) CW *ColE1*	1.7 kb	2.3 kb	1.7 kb
ii) CW Head-on $P_{lacUV5\ 52C}$	2.9 kb	3.4 kb	2.9 kb
iii) CCW Co-directional $P_{lacUV5\ 52C}$	3.9 kb	3.9 kb	4.4 kb
iv) CCW *ColE1*	5.2 kb	5.2 kb	5.7 kb

Supplementary Figure 2

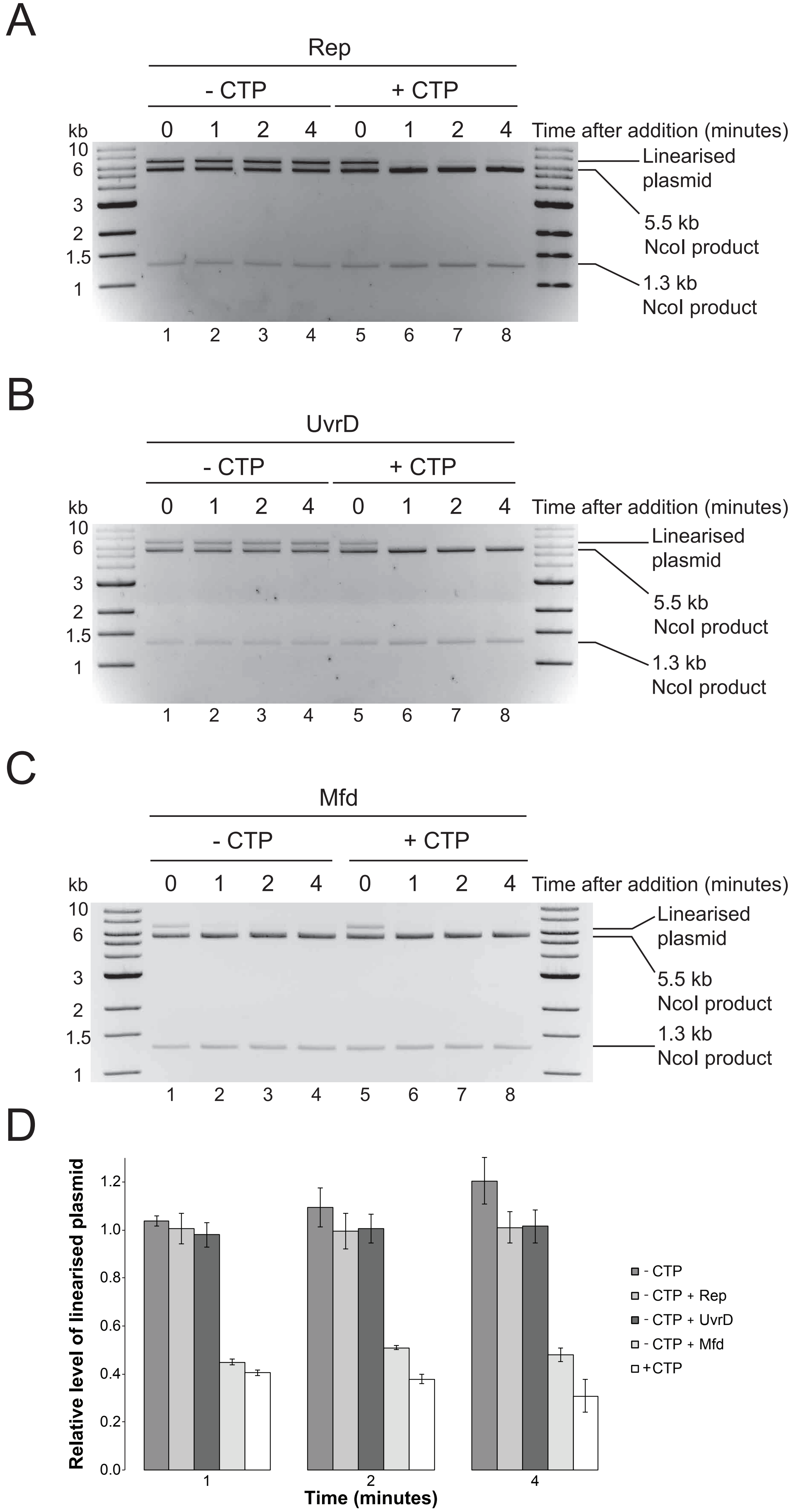

Supplementary Figure 3

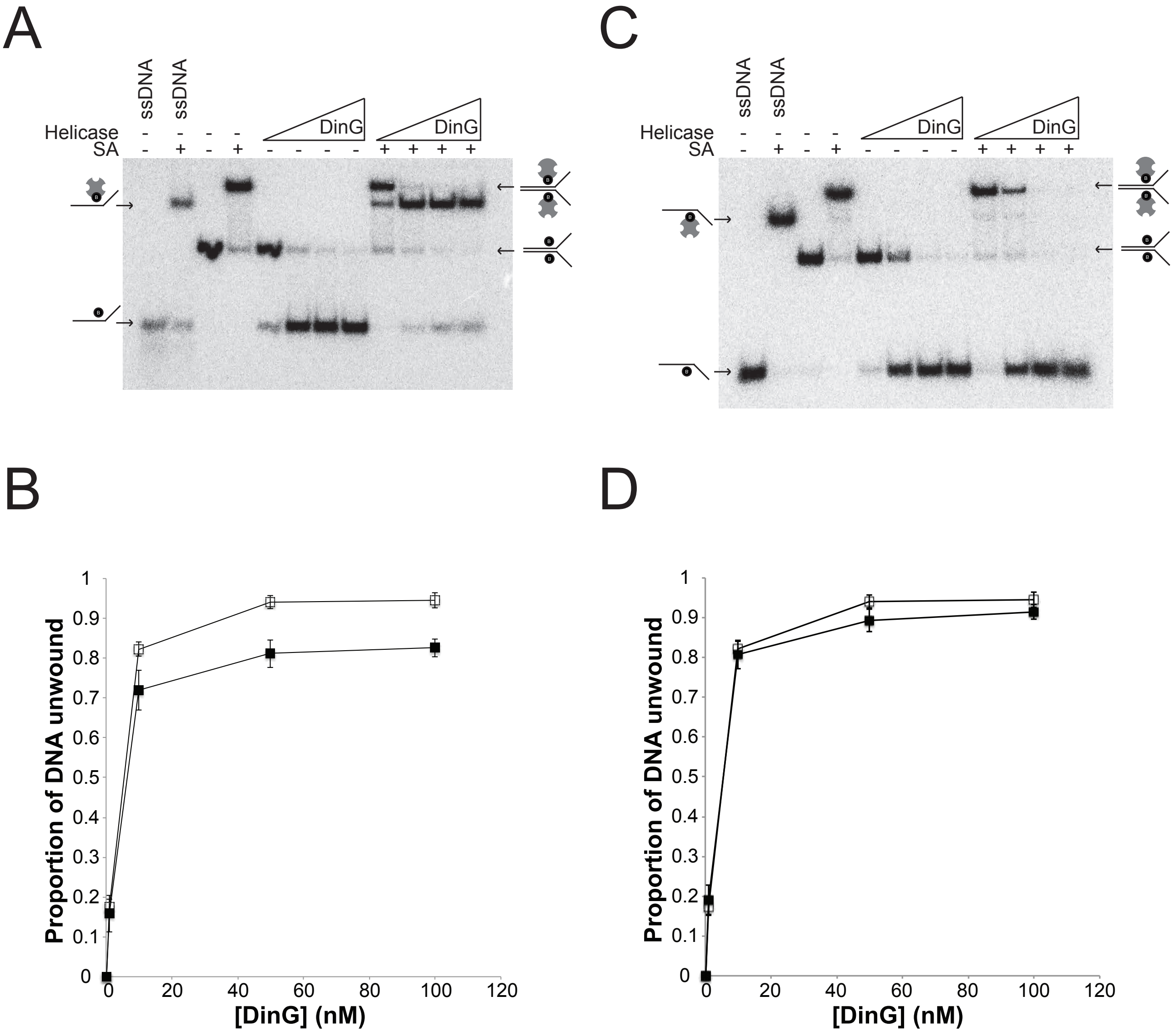

Supplementary Figure 4

Supplementary Tables

Supplementary Table 1: Oligonucleotides

Name	Sequence
oMH046	CGGTGACATATGGCAAAAGT
oMH049	CCACGACATATGCTTGAAATCAATGTTGTATGAATAAGGTTAAAGCCATATTCAACGGGAAAC
oMH070	AGCCATATGCAACGGGAAAC
oMH097	AAATGGTACCCAGTGAGCTCTTTAAATCAATCTAAAGTATATATGAG
oMH098	GAGCTCACTGGGTACCATTTTTAATTTAAAAGGATCTAGGTG
oMH099	AAATGGTACCCACAAAAAACCCCTCAAGACCCGTTTAGAGGCCCCAAGGGGTTATGCTAGGTGAGCTCTTTAAATCAATCTAAAGTATATATGAG
oMH111	GCAATTATCATTGCATCATTCCCCTCGAGTTTTCGAATGAGTTTCTATTATG
oMH162	GAACCGGTACCAAAAATAGTG
oMH163	TGACGAGCTCTTAAAGCG
oMH159	TGACGGTACCCTAGCTTGTGGCGGATAAC
oMH164	TGACGGTACCGAGTCCTGCAATTGTTATCC
oMH188	TGACCGGCCGAATGGTACCGAGTCCTGC
oMH189	TGACCGGCCGTGGCGAGAGAGTGATGGT
oMH119	GTGCTCATCATTGGAAAACGTTC
oMH121	Biotin-CACCAGCTCATACTGGCTGG
PM187B20	GTCGGATCCTCTAGACAGC(biodT)CCATGATCACTGGCACTGGTAGAATTCGGC
PM188B34	AACGTCATAGACGATTACATTGCTACATGGAGC(biodT)GTCTAGAGGATCCGAC

Supplementary Table 2: *Escherichia coli* K-12 strains

Strain number	Relevant Genotype[a]	Source
**MG1655 derivatives**		
MG1655	F^{-} *rph-1*	(8)
HB169	*ΔlacIZYA dinG::<kan>*	TB28 × P1.PM501 (Keio strain JW0784 *ΔdinG::<kan>* (9)) to Km^{r}
JD1141	*ΔlacIZYA oriZ-<cat> Δrep::dhfr*	(10)
JD1277	*ΔlacIZYA oriZ-<cat> dinG::<kan>*	RCe544 × P1.HB169 to Km^{r}
JD1374	*ΔlacIZYA dinG::<>*	HB169 × pCP20 to 37°C Km^{s} Amp^{s}
JD1375	*ΔlacIZYA dinG::<> oriZ-<cat>*	JD1374 × P1.RCe544 to Cm^{r}
JD1376	*ΔlacIZYA dinG::<> oriZ-<cat> pJD001*	JD1375 × pJD001 to Ap^{r}
JD1377	*ΔlacIZYA dinG::<> oriZ-<cat> ΔoriC::kan*[b] *pJD001*	JD1376 × P1.RCe576 to Km^{r}
JD1400	*rpoB*35 dinG::<kan>*	N5925 × P1.HB169 to Km^{r}
JD1401	*rpoB*35 dinG::<>*	JD1400 × pCP20 to 37°C Km^{s} Amp^{s}
JD1402	*rpoB*35 dinG::<> oriZ-<cat> pJD001*	RCe741 × pJD001 to Amp^{r}
JD1404	*rpoB*35 dinG::<> oriZ-<cat> ΔoriC::kan*[b] *pJD001*	JD1402 × P1.RCe576 to Km^{r}
N5925	*rpoB*35 ΔlacIZYA*	(11)
RCe504	*oriZ-<cat>*	(2)
RCe544	*ΔlacIZYA oriZ-<cat>*	(2)
RCe741	*rpoB*35 dinG::<> oriZ-<cat>*	JD1401 × P1.RCe544 to Cm^{r}
TB28	*ΔlacIZYA*	(5)

a – Only the relevant additional genotype of the derivatives is shown. The abbreviations *kan*, *cat* and *dhfr* refer to insertions conferring resistance to kanamycin (Km^{r}), chloramphenicol (Cm^{r}) and trimethoprim (Tm^{r}), respectively. ‘<’ and ‘>’ indicates the use of *frt* sites, where *frt* stands for the 34 bp recognition site of the FLP/*frt* site-directed recombination system. Thus, <*cat*> refers to a kanamycin marker flanked by an *frt* site either side. ‘<>’ indicates loss of the marker in between the *frt* sites following expression of the FLP recombinase from plasmid pCP20 (12).

b – Δ*oriC* refers to a replacement of the entire origin region (754 bp) including DnaA boxes and 13mers as well as the entire *mioC* gene by a kanamycin resistance cassette (13).

Supplementary Information References

1. Bruning, J.G., Howard, J.A. and McGlynn, P. (2016) Use of streptavidin bound to biotinylated DNA structures as model substrates for analysis of nucleoprotein complex disruption by helicases. *Methods*, **108**, 48-55.
2. Ivanova, D., Taylor, T., Smith, S.L., Dimude, J.U., Upton, A.L., Mehrjouy, M.M., Skovgaard, O., Sherratt, D.J., Retkute, R. and Rudolph, C.J. (2015) Shaping the landscape of the *Escherichia coli* chromosome: replication-transcription encounters in cells with an ectopic replication origin. *Nucleic Acids Res.*, **43**, 7865-7877.
3. Skovgaard, O., Bak, M., Lobner-Olesen, A. and Tommerup, N. (2011) Genome-wide detection of chromosomal rearrangements, indels, and mutations in circular chromosomes by short read sequencing. *Genome Res*, **21**, 1388-1393.
4. Muller, C.A., Hawkins, M., Retkute, R., Malla, S., Wilson, R., Blythe, M.J., Nakato, R., Komata, M., Shirahige, K., de Moura, A.P. *et al.* (2014) The dynamics of genome replication using deep sequencing. *Nucleic Acids Res*, **42**, e3.
5. Bernhardt, T.G. and de Boer, P.A. (2004) Screening for synthetic lethal mutants in *Escherichia coli* and identification of EnvC (YibP) as a periplasmic septal ring factor with murein hydrolase activity. *Mol. Microbiol.*, **52**, 1255-1269.
6. Mahdi, A.A., Buckman, C., Harris, L. and Lloyd, R.G. (2006) Rep and PriA helicase activities prevent RecA from provoking unnecessary recombination during replication fork repair. *Genes Dev.*, **20**, 2135-2147.
7. Park, J.S., Marr, M.T. and Roberts, J.W. (2002) *E. coli* transcription repair coupling factor (mfd protein) rescues arrested complexes by promoting forward translocation. *Cell*, **109**, 757-767.
8. Bachmann, B.J. (1996) In Neidhardt, F. C., Curtiss III, R., Ingraham, J. L., Lin, E. C. C., Low, K. B., Magasanik, B., Reznikoff, W. S., Riley, M., Schaechter, M. and Umbarger, H. E. (eds.), *Escherichia coli and Salmonella cellular and molecular biology*. Second ed. ASM Press, Washington, DC, pp. 2460-2488.
9. Baba, T., Ara, T., Hasegawa, M., Takai, Y., Okumura, Y., Baba, M., Datsenko, K.A., Tomita, M., Wanner, B.L. and Mori, H. (2006) Construction of *Escherichia coli* K-12 in-frame, single-gene knockout mutants: the Keio collection. *Mol. Syst. Biol.*, **2**, 2006 0008.
10. Dimude, J.U., Midgley-Smith, S.L. and Rudolph, C.J. (2018) Replication-transcription conflicts trigger extensive DNA degradation in Escherichia coli cells lacking RecBCD. *DNA Repair (Amst)*, **70**, 37-48.
11. Guy, C.P., Atkinson, J., Gupta, M.K., Mahdi, A.A., Gwynn, E.J., Rudolph, C.J., Moon, P.B., van Knippenberg, I.C., Cadman, C.J., Dillingham, M.S. *et al.* (2009) Rep Provides a Second Motor at the Replisome to Promote Duplication of Protein-Bound DNA. *Mol. Cell*, **36**, 654-666.
12. Datsenko, K.A. and Wanner, B.L. (2000) One-step inactivation of chromosomal genes in *Escherichia coli* K-12 using PCR products. *Proc. Natl. Acad. Sci. U S A*, **97**, 6640-6645.

13. Rudolph, C.J., Upton, A.L., Stockum, A., Nieduszynski, C.A. and Lloyd, R.G. (2013) Avoiding chromosome pathology when replication forks collide. *Nature*, **500**, 608-611.
